# Nicotine-induced Genetic and Epigenetic Modifications in Primary Human Amniotic Fluid Stem Cells

**DOI:** 10.2174/0113816128305232240607084420

**Published:** 2024-06-11

**Authors:** Prabin Upadhyaya, Cristina Milillo, Annalisa Bruno, Federico Anaclerio, Carlotta Buccolini, Anastasia Dell’Elice, Ilaria Angilletta, Marco Gatta, Patrizia Ballerini, Ivana Antonucci

**Affiliations:** 1 Center for Advanced Studies and Technology (CAST), “G. d’Annunzio” University of Chieti-Pescara, Chieti 66100, Italy;; 2 Department of Psychological, Health and Territorial Sciences, “G. d’Annunzio” University of Chieti-Pescara, Chieti 66100, Italy;; 3 Department of Innovative Technologies in Medicine & Dentistry, “G. d’Annunzio” University of Chieti-Pescara, Chieti 66100, Italy

**Keywords:** Primary human amniotic fluid stem cells, nicotine, epigenetics, tobacco-related disorders, adipogenesis, mRNAs

## Abstract

**Background:**

Smoking during pregnancy has been linked to adverse health outcomes in offspring, but the underlying mechanisms are not fully understood. To date, the effect of maternal smoking has been tested in primary tissues and animal models, but the scarcity of human tissues limits experimental studies. Evidence regarding smoking-related molecular alteration and gene expression profiles in stem cells is still lacking.

**Methods:**

We developed a cell culture model of human amniotic fluid stem cells (hAFSCs) of nicotine (NIC) exposure to examine the impact of maternal smoking on epigenetic alterations of the fetus.

**Results:**

NIC 0.1 µM (equivalent to “light” smoking, *i.e*., 5 cigarettes/day) did not significantly affect cell viability; however, significant alterations in DNA methylation and N6-methyladenosine (m6A) RNA methylation in hAFSCs occurred. These epigenetic changes may influence the gene expression and function of hAFSCs. Furthermore, NIC exposure caused time-dependent alterations of the expression of pluripotency genes and cell surface markers, suggesting enhanced cell stemness and impaired differentiation potential. Furthermore, NIC-treated cells showed reduced mRNA levels of key adipogenic markers and hypomethylation of the promoter region of the imprinted gene H19 during adipogenic differentiation, potentially suppressing adipo/lipogenesis. Differential expression of 16 miRNAs, with predicted target genes involved in various metabolic pathways and linked to pathological conditions, including cognitive delay and fetal growth retardation, has been detected.

**Conclusion:**

Our findings highlight multi-level effects of NIC on hAFSCs, including epigenetic modifications, altered gene expression, and impaired cellular differentiation, which may contribute to long-term consequences of smoking in pregnancy and its potential impact on offspring health and development.

## 1 INTRODUCTION

Almost 20-30% of the world’s population over 15 years of age smoke tobacco cigarettes [1, 2], including both men and women. Among the female smokers, most of them are of reproductive age. According to the Centers for Disease Control and Prevention (CDC) report, in 2016, 1 in 14 pregnant women smoked during pregnancy in the USA [3]. The amount of nicotine (NIC) in amniotic fluid depends upon factors such as the number of cigarettes smoked per day, the strength of the cigarette, and NIC inhalation mode, *e.g*., traditional cigarettes, roll-your-own cigarettes, electronic cigarettes, cigars, and waterpipes [4, 5].

Several epidemiological studies suggest that prenatal tobacco exposure may be associated with various complications for offspring, including obesity [6], birth defects [7], attention deficit hyperactivity disorder (ADHD) [8], chronic obstructive pulmonary disease [9], asthma [10] and type-2 diabetes [11] during childhood and later life. Recent evidence suggests that the detrimental effects of maternal smoking are correlated with altered DNA methylation [12-14] and dysregulated expression of microRNAs (miRNAs) [15-17]. A large body of preclinical studies conducted on animal models demonstrated smoking-related epigenetic alterations in rats exposed to NIC [17-20]. In particular, recent works in rodents confirmed the direct implication of perinatal NIC exposure on early adipogenesis and lipogenesis, resulting in increased offspring adiposity [21-23]. Furthermore, in the last few years, a growing number of studies have systematically investigated smoking-related molecular alteration and gene expression profiles in stem cells [24-26]. Stem cells and their capability to differentiate and repair organs damaged by smoking are involved in diseases associated with tobacco use. Given this evidence, human amniotic fluid stem cells (hAFSCs) provide an *in vitro* model for studying epigenetic regulation in early human development [27]. Thus, hAFSCs could represent an interesting alternative to induced pluripotent stem cells (iPSCs) for identifying epigenetic marks in diseased gestation. In our previous experiments, we obtained hAFSCs from the second trimester of the amniotic fluid [28, 29]. Given their characteristic of fetal stem cells, hAFSCs are the ideal candidate for *in vitro* studies concerning the effects of NIC on the fetus. In the present study, we differentiated hAFSCs into adipogenic lineage to elucidate the epigenetic effects of perinatal NIC exposure on early adipogenesis. The clarification of molecular changes induced by maternal smoking may have important implications for identifying potential biomarkers predictive of tobacco-related disorder development during prenatal and postpartum periods.

## 2 MATERIALS AND METHODS

### 2.1 hAFSC Culture

The samples of human amniotic fluid (2-3 ml) were obtained from women during amniocentesis (n=5) for prenatal diagnosis (16-18 weeks of pregnancy) at SS Annunziata Hospital, ASL Lanciano-Chieti-Vasto, Chieti, Italy. The study was carried out following the recommendations of the Declaration of Helsinki. An informed consent was obtained from each subject. After collection, amniotic fluid samples were centrifuged at 1200 rpm for 5 minutes, and the pellet was utilized to establish the cell line. hAFSCs were cultured until the fifth passage as previously described [29, 30].

### 2.2 Nicotine Preparation

NIC was freshly prepared for each treatment. The liquid form of NIC (≥ 99% (GC), liquid, N3876) was purchased from Sigma Aldrich SRL (Milan, Italy). From the liquid NIC, a stock solution of 100 mM was prepared by diluting 16 µL of NIC in 984 µL of phosphate-buffered saline (PBS). From the stock, a NIC solution of 1000 µM was prepared by diluting 10 µL of stock solution in 990 µL of culture medium. With this solution, NIC 0.1μM for experiments was obtained by serial dilution.

### 2.3 Cell Viability

The 3-(4,5-dimethylthiazol-2-yl)-5-(3-carboxymethoxy- phenyl)-2-(4-sulfophenyl)-2H-tetrazolium (MTS) assay was performed to evaluate cell proliferation in response to NIC treatment by using CellTiter 96^®^ AQueous One Solution Cell Proliferation Assay (Promega Italia s.r.l., Milan, Italy), following the manufacturer’s protocol, as previously described [31-34]. Briefly, the cells were seeded at 3000 cells/well and incubated for 24 hours at 37°C in a humidified atmosphere (95%) under 5% CO_2_. Furthermore, the cells were treated with NIC 0.1 μM and incubated at 37°C with 5% CO_2_ for 24, 48, and 72 hours, respectively. At each time point, the cell viability was assessed as previously described [29].

### 2.4 Adipogenic Differentiation of hAFSCs

For adipogenic differentiation, once hAFSCs have reached the confluence of 90%, the medium was replaced with the adipogenic differentiation medium (AdipoMAX Differentiation Medium from Sigma-Aldrich by Merck, Darmstadt, Germany). The cells were left to differentiate for 21 days in the absence or the presence of NIC 0.1 μM for the same period.

### 2.5 Extraction of DNA and Total RNA

The extraction of total DNA was performed by using the MagPurix Forensic DNA Extraction Kit (MagPurix^®^, Zinexts Life Science, Taiwan) and the automatic DNA extractor (MagPurix^®^, Zinexts Life Science, Taiwan) according to the manufacturer’s protocol. The extracted DNA was then quantified using the Qubit DNA assay kit (Life Technologies, ThermoFisher Scientific). The total RNA was extracted using acid guanidine thiocyanate phenol-chloroform protocol and was quantified using the Qubit RNA assay kit (Life Technologies, ThermoFisher Scientific). DNA and RNA quantifications were obtained using a Qubit 3.0 fluorometer (ThermoFisher Scientific).

### 2.6 Reverse Transcription and Real-time Quantitative PCR

Total RNA (100 ng) was reverse transcribed into cDNA using RevertAid First Strand cDNA Synthesis Kit (ThermoFisher Scientific) and oligo (dT) primers, following the manufacturer's protocols, obtaining 20 μL of cDNA for each sample. Moreover, to perform real-time quantitative PCR, the primers for C-Kit (KIT proto-oncogene, receptor tyrosine kinase), Oct-4 (POU class 5 homeobox 1), SOX2 (SRY-box transcription factor 2), NANOG (Nanog homeobox), LPL (lipoprotein lipase), PPARG (peroxisome proliferator-activated receptor gamma), FABP4 (fatty acid binding protein 4), and GAPDH (glyceraldehyde-3-phosphate dehydrogenase) were purchased from Eurofins Genomics (Ebersberg, Germany) and are summarized in Table **S1**. The real-time qPCR was performed as previously described [29].

### 2.7 Immunophenotyping with Flow Cytometry

Both primary conjugated and unconjugated anti-human monoclonal antibodies (IgG) against 19 different proteins have been used for flow cytometry. Fluorescently tagged secondary antibodies were used to bind against the primary unconjugated antibodies (Table **S2**). hAFSCs immunophenotyping was performed as previously described [29].

### 2.8 Analysis of Methylation Profiles

Global DNA methylation quantification was performed as previously described [29]. N6-methyladenosine (m6A) RNA methylation quantification was performed on total RNA (200 ng) with the help of EpiQuik™ m6A RNA Methylation Quantification Kit (Epigentek Group Inc.). The amount of m6A in the different samples was calculated in terms of m6A% (m6A/A×100%).

### 2.9 Bisulfite Conversion and Pyrosequencing

Bisulfite conversion and pyrosequencing were performed as previously described [29]. Primers and PCR conditions used in pyrosequencing are described in Table **S3**. The results of pyrosequencing were displayed as a pyrogram. The methylation percentage was expressed for each DNA locus as %5-mC divided by the sum of methylated and unmethylated cytosines.

### 2.10 MicroRNA Profiling and Data Analysis

The cells were allowed to differentiate into Adipogenic lineage for 20 days in two different dishes. During adipogenesis, one dish was treated with NIC 0.1 µM (treatment), and another was left untreated (control). After 20 days of differentiation, total RNA was extracted from both the control and treatment using the acid guanidine-thiocyanate-phenol-chloroform protocol. The total RNAs were sent for the next-generation sequencing experiments, comprising quality control samples, and were performed by Genomix4life S.R.L. (Salerno, Italy). Indexed libraries were prepared from 500 µg of total RNA purified with TruSeq SmallRNA Sample Prep Kit (Illumina, San Diego, CA, USA) according to the manufacturer’s instructions. The libraries were quantified using the Agilent 4200 TapeStation (Agilent Technologies, Rome, Italy) and pooled in such a way that each index-tagged sample was present in equimolar amounts, with the final concentration of the pooled samples being 2 nM. The pooled samples were subject to cluster generation and sequencing using an Illumina NextSeq 500 System (Illumina) in a 1x75 single read format at a final concentration of 3 pM. The raw sequence files generated (fastq files) underwent quality control analysis using FastQC (http://www.bioinformatics.babraham.ac.uk/projects/fastqc/). Bioinformatics analyses were performed with iSmaRT. Starting from raw sequencing data, iSmaRT first conducts quality control and filtering of the sequence reads using FastQC, while Cutadapt [35] or sRNAbench [36] is used to remove the adapter sequences and low-quality reads. The differential expression analysis is performed in iSmaRT integrating three Bioconductor statistical packages: DESeq2 [37], edgeR [38], and NOISeq [39]. The expression of a total of 1018 miRNAs was quantified using this technique (Table **S4**). The miRNAs with more than 7 reads were considered expressed. We invented simple cut-offs to classify the expression into four distinct categories. The miRNAs with ≥ 10,000 reads were considered highly expressed, miRNAs with 9999-1000 reads were considered highly expressed, miRNAs with 999-100 reads were considered moderately expressed, and miRNAs with 99-8 reads were considered low expressed.

### 2.11 Statistical Analysis

For each sample, technical duplicates were performed, and their averages were used for data interpretation. For each experiment averages from 5 different hAFSC lines were obtained and analyzed using Graph Pad Prism V6 (California, USA). Multiple t-tests were performed to assess statistical significance without corrections for multiple comparisons. Data are presented as mean ± SD, n=3-6, as specified in each figure legend. *P*-values were expressed as **** when *P ≤* 0.0001, *** when *P ≤* 0.001, ** when *P ≤* 0.01, and * when *P ≤* 0.05. The *P* value > 0.05 indicates no statistical significance (ns).

## 3 RESULTS

### 3.1 Effect of Nicotine on hAFSC Viability

NIC concentration in the amniotic fluid of smoking pregnant women (5 cigarettes/day) has been reported to be in the range of 7-31 ng/ml, with a median of 11 ng/ml (0.07 µM) [40, 41]. Thus, hAFSCs were treated with NIC (0.1 μM) for 24, 48, and 72 hours, and cell viability was assessed by MTS assay. Cell exposure to NIC 0.1 μM only slightly and not significantly affect hAFSCs viability up to 72 hours as shown in Fig. (**1**).

### 3.2 Nicotine Increases Global DNA Methylation and Induces N6-methyladenine Modifications in hAFSCs

**Fig. (2):**
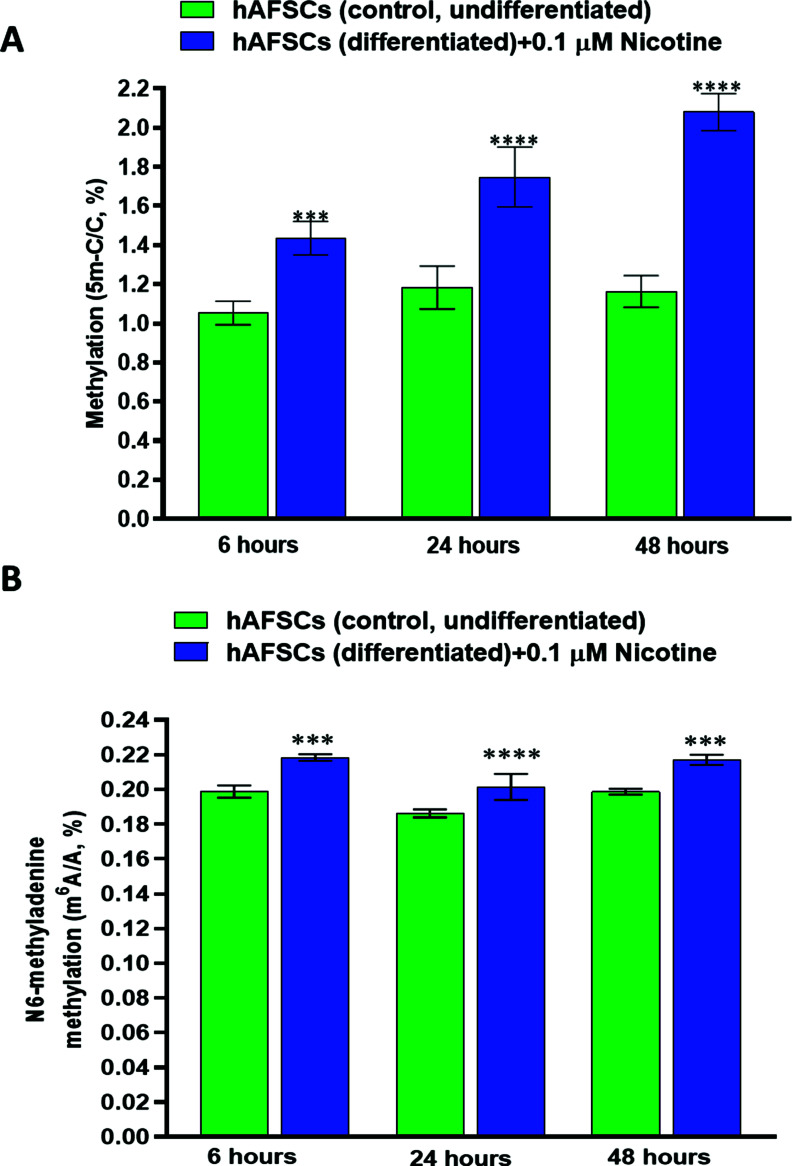
(**A**) Effects of nicotine (NIC) treatment on global DNA methylation (5 m-C) of hAFSCs (undifferentiated). hAFSCs (3000 cells/well) were incubated for 24 hours at 37°C in a humidified atmosphere (95%) under 5% CO_2_. Then, the cells were treated with Nicotine 0.1 μM and incubated at 37°C in a humidified atmosphere with 5% CO_2_ for 6, 24, and 48 hours respectively. Global DNA methylation quantification was assessed and reported, at each time point, as 5 mC/C ratios (%). At each time point, data are reported as mean ± SD, n=3 (each analyzed in duplicate). *** *≤* 0.001 and **** *≤* 0.0001 *vs.* control (untreated cells) at the same time point. (**B**) The effects of Nicotine (NIC) treatment on N6-methyladenine (m^6^A) modification of hAFSCs (undifferentiated). hAFSCs (3000 cells/well) were incubated for 24 hours at 37°C in a humidified atmosphere (95%) under 5% CO_2_. Then, the cells were treated with NIC 0.1 μM and incubated at 37°C in a humidified atmosphere with 5% CO_2_ for 6, 24, and 48 hours, respectively. m^6^A methylation was assessed as m^6^A/A ratios (%). Data are reported as mean ± SD, n = 3 (each analyzed in duplicate). ***P ≤ 0.001 and ****P ≤ 0.0001 *vs.* control (untreated cells) at the same time point.

DNA methylation profiles play an important role during embryogenesis and the early development of the fetus. Thus, we evaluated the effect of NIC treatment on the extent of DNA methylation at 6, 24, and 48 hours. Our results indicate a time-dependent increase of DNA methylation at the fifth position of cytosine (5 m-C/C %) in the hAFSCs exposed to NIC (0.1 µM). As expected, DNA methylation was not modified over time in untreated cells (Fig. **2A**). The highest methylation level was observed after 48 hours of cell exposure to NIC (2.08 ± 0.09%, (mean ± SD) *vs.* 1.05 ± 06% for NIC-treated and untreated hAFSCs, respectively).

N6-methyladenine (m6A) modification is increasingly recognized as one of the post-transcriptional key markers in different types of RNAs as well as one critical factor in the regulation of RNA splicing, translation, stability, and translocation [42-44]. Therefore, we also evaluated the effect of NIC on m6A methylation level in RNA extracted from hAFSCs exposed to the drug for 6, 24, and 48 hours. Fig. (**2B**) shows a slight but significant increase in the percentage of m6A methylation in hAFSCs treated with NIC 0.1 µM at all time points compared to the untreated cells. The rate of m6A methylation was significantly enhanced already at 6 hours of NIC treatment (0.22 ± 0.001% *vs.* 0.199 ± 0.003% (mean ± SD) for NIC-treated *vs.* untreated cells) and remained almost stable up to 48 hours.

### 3.3 Nicotine Alters the Expression of Pluripotency Genes and Cell Surface Protein Markers in hAFSCs

**Fig. (3):**
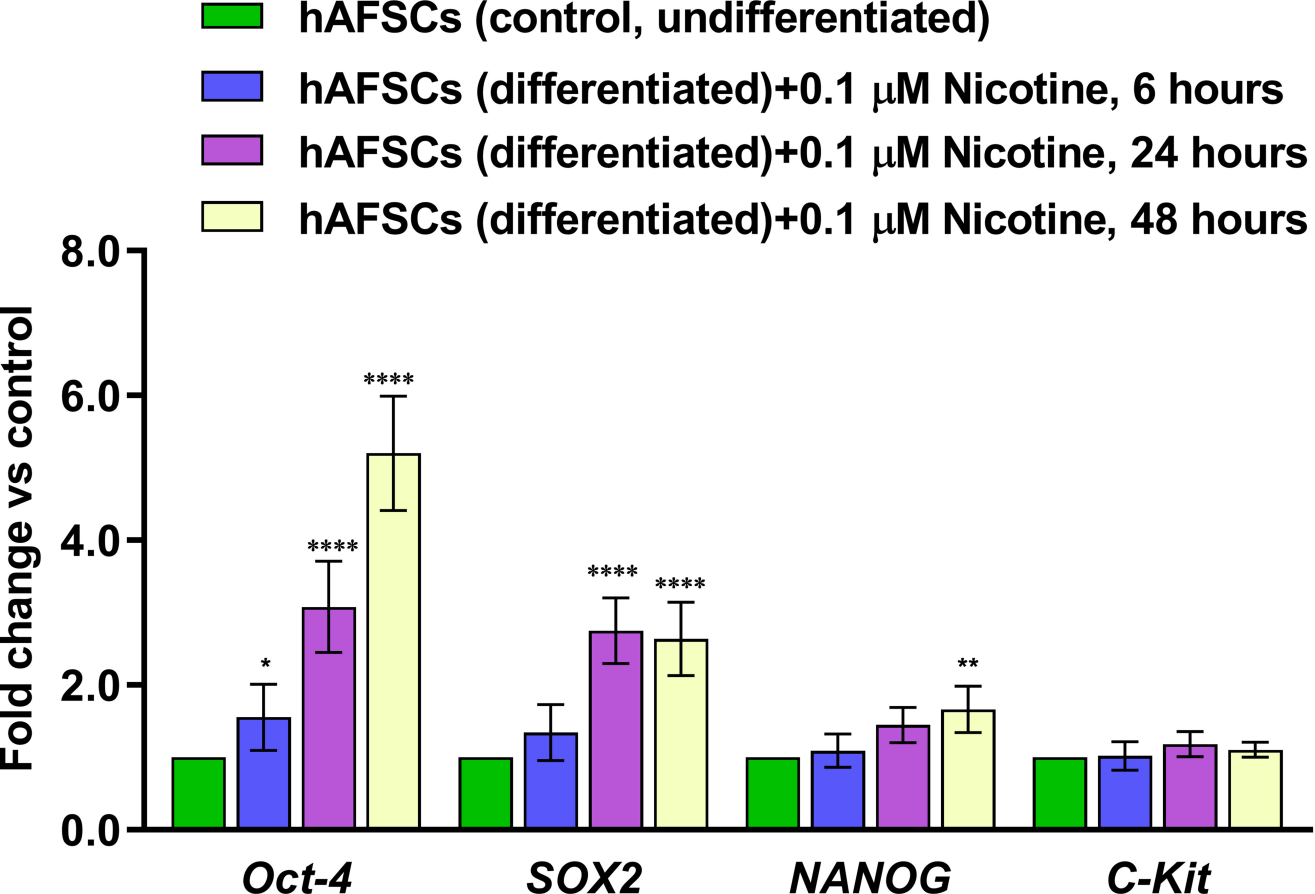
The effects of nicotine (NIC) treatment on the expression of pluripotency markers of hAFSCs (undifferentiated). hAFSCs (3000 cells/well) were incubated for 24 hours at 37°C in a humidified atmosphere (95%) under 5% CO_2_. Then, the cells were treated with NIC 0.1 μM and incubated at 37°C in a humidified atmosphere with 5% CO_2_ for 6, 24, and 48 hours respectively. The gene expression of Oct-4 (POU class 5 homeobox 1), SOX2 (SRY-box transcription factor 2), NANOG (Nanog homeobox), and C-Kit (KIT proto-oncogene, receptor tyrosine kinase) was evaluated by qPCR and normalized to those of GAPDH (glyceraldehyde-3-phosphate dehydrogenase) and reported as fold-change *vs.* the gene expression detected in control samples. Data are reported as mean ± SD, n = 5 (each analyzed in duplicate) *P ≤ 0.05, **P ≤ 0.01; and ****P ≤ 0.0001 *vs.* control (untreated cells) at the same time point.

Furthermore, to delineate the impact of NIC on the differentiation potential of hAFSCs, we assessed the expression pattern of pluripotency genes, such as Oct-4, SOX2, NANOG, and C-Kit [45] in NIC-treated hAFSCs, compared with untreated cells. NIC 0.1 µM significantly increased the expression levels of Oct-4, SOX2, and NANOG, but not of C-Kit, after 48 hours of exposure (Fig. **3**). Only Oct-4 expression was significantly up-regulated as early as after 6 hours of drug exposure. These results support the hypothesis that NIC exposure can also promote an enhancement of stemness in hAFSCs with a potential negative impact on differentiation processes.

We also assessed the expression profile of the principal mesenchymal markers in hAFSCs (Table **1**). As previously reported [46-48], the cells were negative for hematopoietic markers (*e.g*., CD14, CD34, CD45) and positive for various mesenchymal markers (*e.g*., CD73, CD90, CD105), as well as for related surface adhesion molecules (*e.g*., CD29, CD44, CD146, CD166) (Table **1**). Interestingly, NIC treatment did not affect these markers except for CD13 and CD146, which resulted in them being significantly up- and down-regulated, respectively (Table **1**).

### 3.4 Nicotine Reduced Adipogenic Differentiation of hAFSCs and Alters the Methylation Status of Imprinted Gene H19 in Differentiated hAFSCs

**Fig. (4):**
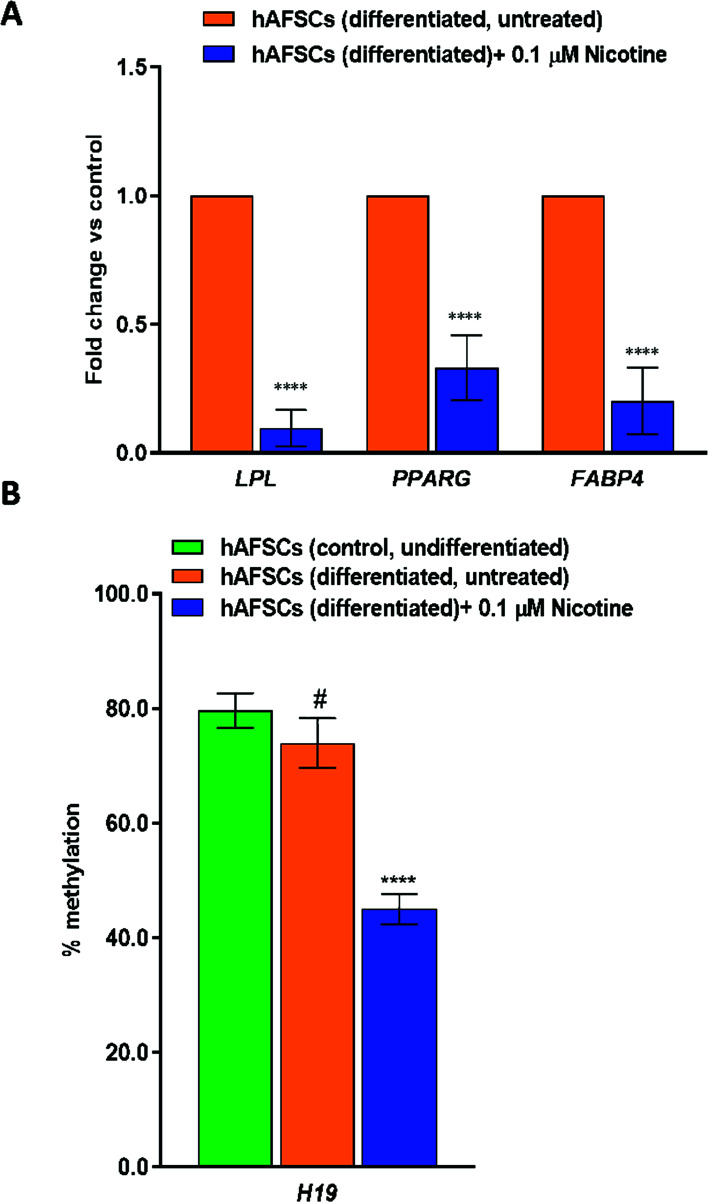
(**A**) The effects of NIC treatment on the expression of adipogenic markers in hAFSCs (differentiated). In addition, to induce adipogenic differentiation, once hAFSCs have reached the confluence of 90%, the medium was replaced with the adipogenic differentiation medium (AdipoMAX Differentiation Medium). The cells were left to differentiate for 21 days in the absence (control) or in the presence of Nicotine (NIC) 0.1 μM (treated) for the same time period. The gene expression of LPL (lipoprotein lipase), PPARG (peroxisome proliferator-activated receptor gamma), and FABP4 (fatty acid binding protein 4) was evaluated by qPCR and normalized to those of GAPDH and reported as fold-change *vs.* the gene expression detected in control samples. Data are reported as mean ± SD, n=3 (each analyzed in duplicate). ****P ≤ 0.0001 *vs.* control (untreated cells). (**B**) Promoter-specific methylation change in control (untreated), differentiated nicotine (NIC) 0.1 µM treated (21 days), and untreated cells (21 days). Data are reported as mean ± SD n = 3 (each analyzed in duplicate). ****P ≤ 0.0001 *vs.* hAFSCs (undifferentiated) and # *P ≤* 0.0001 *vs.* hAFSCs (untreated).

Moreover, to investigate the effect of NIC on adipogenesis, hAFSCs were differentiated into adipogenic lineage in the absence and the presence of NIC 0.1 µM, and the expression of key adipogenic markers such as LPL, PPARG and FABP4 was evaluated during the terminal differentiation stage. Real-time qPCR analysis revealed a significant decrease in the mRNA levels of LPL, PPARG, and FABP4 induced by NIC (Fig. **4A**), with LPL showing the greatest reduction (0.097 ± 0.071; *P* < 0.0001), followed by FABP4 (0.202 ± 0.129; *P* < 0.0001) and PPARG (0.331 ± 0.126; *P* < 0.0001), respectively (Fig. **4A**).

It is widely recognized that H19, an imprinted long non-coding RNA (lncRNA), plays a crucial role in lipid metabolism [49], and recent data have indicated that maternal smoking has an important impact on methylation levels of H19 in fetuses [50-52]. Thus, in our study, we investigated the DNA methylation pattern of the H19 gene in NIC-treated differentiated hAFSCs. Compared with the control, we observed a marked hypomethylation in the promoter region of H19 of differentiated cells treated with NIC 0.1 µM (Fig. **4B**).

### 3.5 Change in the Expression of miRNAs During Nicotine Treatment

**Fig. (5):**
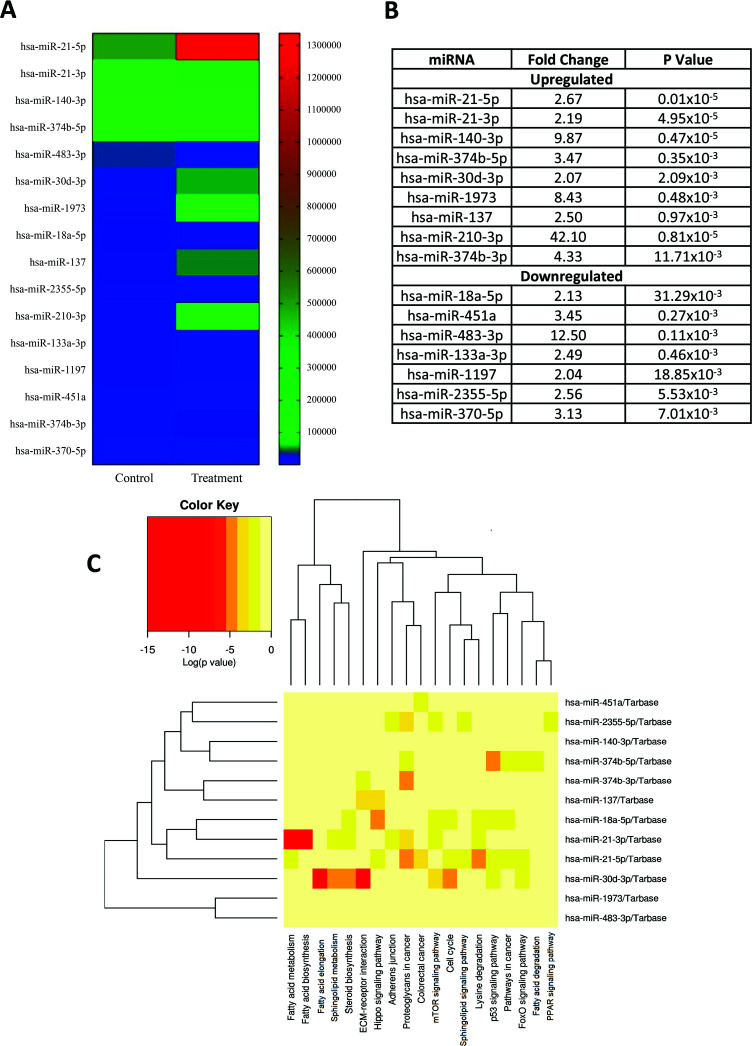
(**A** and **B**) Significant changes of miRNA expression in NIC 0.1 µM treated cells (differentiated) for 21 days by using Next Generation approach (Illumina platform). Fold change ≥ 2 are included in the study (n=5). (**C**) Pathway prediction of miRNAs differentially expressed between treated differentiated hAFSCs and untreated differentiated cells. Red colour shows the most significant pathway involving each miRNA.

Moreover, to study the impact of NIC on miRNAs involved in adipogenic differentiation, we employed the Next Generation approach utilizing the Illumina platform. The miRNA expression levels were compared between NIC-treated differentiated hAFSCs and untreated differentiated cells. In an initial analysis of 1018 miRNAs (Table **S4**), 442 were qualified for evaluation, with more than 7 reads. Out of these, 28 had very high expression (≥ 10,000 reads) (Table **S5**), 64 were highly expressed (9999-1000 reads) (Table **S6**), 131 were moderately expressed (999-100 reads) (Table **S7**), and 219 were lowly expressed (99-8 reads) (Table **S8**). Using a fold change threshold of ≥ 2 and a P-value threshold of ≤ 0.05, we found 16 differentially expressed miRNAs (Fig. **5A**), with 7 being down-regulated and 9 being up-regulated (Fig. **5B**). As shown in Fig. (**5**), miR-210-3p (fold change = 42.10) and hsa-miR-483-3p (fold change = 12.50) were the most highly up-and-down-regulated miRNAs, respectively.

In addition, to identify the biological function of the differentially expressed miRNAs, the DIANA software and miRNet were used to predict the potential target pathways and genes for all up- regulated and down-regulated miRNAs. In a total of 16 differentially expressed miRNA, four (hsa-miR-133a-3p, hsa-miR-1197, hsa-miR-370-5p, and hsa-miR-210-3p) were not recognized by Diana Tools and were consequently removed from the analysis. The remaining 12 miRNAs were analyzed by KEGG analysis to predict the signaling pathways with a criterion of *P <* 0.05 for selecting significance. In total, 59 pathways were detected, and only 12 were involved in fatty acid metabolism, fatty acid biosynthesis, fatty acid elongation, fatty acid degradation, signaling pathways regulating pluripotency of stem cells, pathways in cancer and sphingolipid metabolism (Table **2**), as illustrated by the heat map (Fig. **5C**).

The functional enrichment analysis for the predicted target genes of 16 dysregulated miRNAs was performed using the bioinformatics tool miRNet at a *P*-value of 0.05. A total of 7758 targeted genes were identified, including 6595 genes for upregulated miRNAs and 2944 genes for downregulated miRNAs (Fig. **S1**). The target genes of 16 miRNAs differentially expressed were particularly enriched in glycerophospholipid metabolism, mTOR signaling pathway, sphingolipid metabolism, fatty acid metabolism, and glycerolipid metabolism (Figs. **S2A**, **B**). Furthermore, DisGeNet analysis, a disease database, was used to predict diseases associated with miRNA-target gene. Several miRNAs were significantly involved in cognitive delay, intrauterine retardation, infant small for gestational age, fetal growth retardation, failure to gain weight, and premature birth (Figs. **S3A**, **B**). Our findings pointed out the important role of miRNAs in fatty acid biosynthesis and, more interestingly, showed that their predicted target genes are mainly involved in fetal growth and intrauterine retardation birth, according to the DisGeNet database.

## 4 DISCUSSION

There is remarkable evidence confirming the harmful effects of maternal smoking during pregnancy on the fetus, partly caused by epigenetic alterations [53-55]. Research is ongoing to better understand the epigenetic machinery underlying diseases associated with prenatal NIC exposure. In this context, stem cells represent a promising system for modeling fetal toxicology. In this study, we investigated the epigenetic effect of NIC on the adipogenic differentiation of hAFSCs as a cellular culture model of prenatal tobacco exposure. The advantages of hAFSCs include their properties of plasticity intermediate between embryonic and adult stem cells and their capacity to differentiate into several cell lineages [30, 56-60]. It is well-recognized that cigarette smoking during pregnancy has dose-dependent perinatal outcomes [61]. In our experiments, we used NIC at the concentration of 0.1 µM, which was within the range of smoking pregnant women defined as “light” smokers (5 cigarettes/day) [40, 41]. We found that NIC 0.1 μM did not induce significant changes in cell viability. This is consistent with previous studies reporting no significant effects on overall cell survival for low concentrations of NIC (0.1-10 μM) [25, 62]. However, our *in vitro* model with hAFSCs revealed that maternal smoking during pregnancy may significantly alter fetal DNA methylation.

We measured the DNA methylation and m6A methylation levels of hAFSCs exposed to NIC, and significant increases in both types of methylation compared to untreated cells were found. Emerging evidence has reported that the m6A modifications play a critical role in the development of stem cells, including self-renewal and differentiation [63-65]. In particular, m6A modification is highly involved in regulating adipogenesis and the progression of human metabolic disease [66-68]. In addition, several studies have described that increased m6A levels might alter the normal differentiation pathway, resulting in overexpression of genes associated with pluripotency [69-71]. Thus, we monitored the expression pattern of pluripotency genes and cell surface markers and found that NIC altered their expression in a time-dependent manner. NIC increased the expression of Oct-4, SOX2, and NANOG after 48 hours of exposure, indicating that NIC enhanced the stemness of treated cells and, therefore, may impair their differentiation potential. Previous studies have also suggested that cigarette smoke can affect the stemness of exposed cells [72, 73]. Moreover, the findings that CD13 is upregulated, while CD146 is downregulated in NIC-treated cells compared to the control cells provide additional evidence of a significant role of NIC in the inflammatory process. It has been previously reported that smoking is associated with systemic inflammation and elevated levels of circulating CD13-positive immunosuppressive cells [74]. On the other hand, the expression of endothelial adhesion molecule CD146 was significantly low in the treated cells. Accordingly, Kratzer and co-workers [75] found that the treatment of rat pulmonary microvascular endothelial cells with cigarette smoke extract decreased both the gene and protein expression of CD146. Cigarette smoke extract contains a complex cigarette mixture of over 7,000 chemicals [76]. Our *in vitro* study suggests that NIC is at least one of the components that could cause the downregulation of CD146.

It is well recognized that NIC exposure may significantly affect the metabolic function of adipose tissue, however, results from studies addressing the harmful effects of NIC on adipogenic differentiation are controversial. Wahl and colleagues demonstrated that cigarette smoking or nicotine did not affect the adipogenic differentiation capacity of human mesenchymal stem cells [77]. Differently, periodontal ligament-derived stem cells from cigarette smokers produce higher lipid levels than non-smokers [78], while Zagoriti *et al*. [79] found that cigarette smoking impaired the differentiation of pre-adipocytes to beige adipocytes. In line with these recent findings, our experiments demonstrate that low NIC concentrations down-regulated expression of adipogenic-related genes (LPL, PPARG, and FABP4) in treated hAFSCs, suggesting that NIC inhibited adipogenic differentiation during fetal development. The great variability of results about the adipogenic response to smoking could be related to different factors, such as NIC concentrations, various cell sources, and culture conditions.

Adipogenic impairment is one of the major causes of metabolic syndrome, a class of medical disorders associated with an increased risk for coronary heart disease, cardiovascular atherosclerotic diseases, and type 2 diabetes mellitus, and several crucial aspects of adipogenesis are controlled by epigenetic events [80]. Our results demonstrate a significant hypomethylation in the promoter region of H19 during adipogenic differentiation of the treated cells compared to the control, thus suggesting a role of H19 in the suppression of adipo/lipogenesis. Several evidence support the role of the H19 gene in lipid metabolism and growth regulation during embryonic development, which is widely documented [81-84] and, particularly, in the suppression of adipo/lipogenesis [85-87]. Recently, Zhu and colleagues [88] reported that depletion of human circular RNA H19 increased the expression levels of genes related to lipogenesis, such as CEBPA (CCAAT enhancer binding protein alpha), PPARG, SREBF1c (sterol regulatory element binding transcription factor 1c), FABP4, ACC-1 (acetyl-CoA carboxylase 1), LPL and FAS. Accordingly, we observed hypomethylation of the H19 gene associated with the downregulation of adipogenic markers, including PPARG, FABP4, and LPL. Altogether, these results suggest that NIC could alter the expression levels of H19 through dysregulation of the DNA methylation status, with possible clinical implications correlated with perturbation of fetal growth and metabolic disorders in adulthood.

Rapid advances in the epigenetic field in recent years have demonstrated the role of miRNAs in several diseases, particularly in the prevention, early diagnosis, and prognosis [89]. In the present investigation, a total of 16 differentially expressed miRNAs were detected in treated differentiated hAFSCs, including 7 down-regulated and 9 up-regulated ones. The most up-regulated miRNA was hsa-miR-210-3p (fold change = 42.10), and the most down-regulated miRNA was hsa-miR-483-3p (fold change = 12.50). Recently, Yang *et al*. [90] provided evidence that overexpression of miR-210 suppresses adipogenic differentiation with a significant reduction of adipogenic marker genes PPARG and LPL. Accordingly, we speculated that NIC increases the expression of hsa-miR-210-3p, resulting in the inhibition of adipogenic differentiation *in vitro* of treated differentiated hAFSCs. On the other hand, it has been reported that the downregulation of miR-483-3p inhibits adipogenic differentiation and promotes the proliferation of stem cells [91]. Our data suggest that exposure to NIC induces dysregulation of miRNA involved in the adipogenic process by inhibiting target genes of differentiation. A limitation of this study is that experimental assays to confirm the deregulation and the functional role of the identified miRNAs have not been performed due to the decreased number of pregnant patients undergoing amniocentesis for prenatal diagnosis. This invasive test has been progressively replaced by the development of non-invasive methods of prenatal testing (NIPT) involving the identification of fetal DNA from maternal blood [92]. In this context, testing in animal models resembling miRNA regulation in humans should be required to translate miRNA profiling into clinical relevance as biomarkers of long-term consequences of smoking in pregnancy and its potential impact on offspring health and development.

Finally, the KEGG analysis of the 16 dysregulated miRNAs revealed important pathways mainly involved in fatty acid metabolism and biosynthesis, pluripotency of stem cells, cancer, and sphingolipid metabolism. Subsequently, the gene target analysis showed that the selected miRNAs were able to modulate the expression of genes directly related to glycerophospholipid metabolism, mTOR signaling pathway, sphingolipid metabolism, fatty acid metabolism, and glycerolipid metabolism. Additionally, we performed DisGeNet analyses to predict potential miRNA-disease associations. Interestingly, dysregulated miRNAs showed significant associations with pathologies such as cognitive delay, intrauterine retardation, premature birth, fetal growth retardation, failure to gain weight, and premature birth.

## CONCLUSION

In conclusion, by using a promising hAFSC culture model of NIC exposure, this study showed that NIC alters the properties of the cells at epigenetic, transcriptional, and cellular levels during fetal development. Specifically, NIC increases the DNA and m6A methylation levels, enhances the stemness, and impairs the adipogenic differentiation of hAFSCs. These effects may involve NIC-induced dysregulation of miRNAs inhibiting target genes of adipogenic differentiation. Although these findings need further validation, they provide exploratory, hypothesis-generating results for studying the effects of aberrant epigenetic changes associated with prenatal NIC exposure to improve the understanding of the impact of perinatal maternal smoking on tobacco-related disorders and to develop novel biomarkers for precise diagnosis and future therapies.

## Figures and Tables

**Fig. (1):**
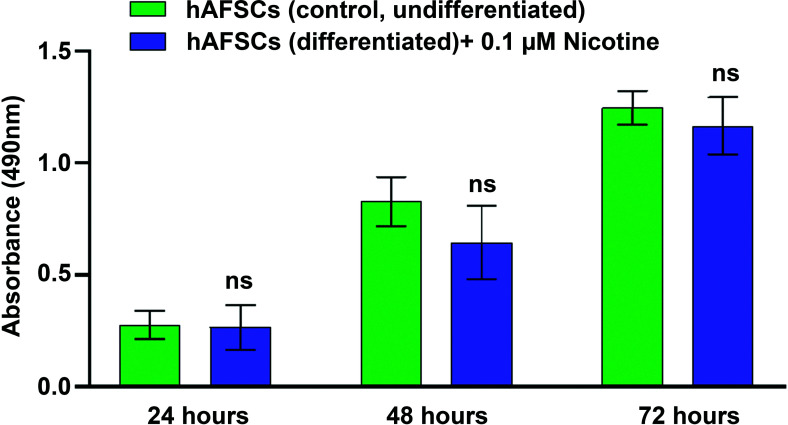
The evaluation of cell viability in hAFSCs after treatment with Nicotine (NIC). hAFSCs (3000 cells/well) were incubated for 24 hours at 37°C in a humidified atmosphere (95%) under 5% CO_2_. Then, cells were treated with NIC 0.1 μM and incubated at 37°C in a humidified atmosphere with 5% CO_2_ for 24, 48, and 72 hours respectively. At each time point, the cell viability was assessed. Background absorbance (490 nm) was subtracted from each data point using a set of wells containing only Iscove's Modified Dulbecco's Medium (IMDM). Data are reported as mean ± SD n = at least 6 (at each time point); “ns” indicates not significant (*P >* 0.05).

**Table 1:** Protein markers used in flow cytometry.

**Markers**	**Control (Untreated)**		**Nicotine 0.1 µM**	
	**(Mean ± SD)**	**Phenotype**	**(Mean ± SD)**	**Phenotype**
CD29	63.7 ± 10.1	++++	81.3 ± 12.6	++++
CD44	55.3 ± 11	++++	63.1 ± 11.2	++++
HLA ABC	45.7 ± 9.9	++++	45.6 ± 9.2	++++
CD73	16.8 ± 6.2	++	14.7 ± 7.1	++
*CD146	35.8 ± 12.7	+++	15.7 ± 7.6	++
CD166	12.8 ± 3.9	++	12 ± 4.4	++
CD90	6.5 ± 2.7	+	3.7 ± 1.1	+
*CD13	3.9 ± 1.8	+	17.4 ± 3.8	++
CD105	2.2 ± 0.9	+/-	2 ± 1.2	-
CD144	1.4 ± 0.2	-	1 ± 0.3	-
HLA DR	1.2 ± 0.1	-	1.2 ± 0.1	-
CD14	1.2 ± 0.2	-	1.2 ± 0.1	-
CD34	1.1 ± 0.1	-	1.9 ± 0.2	-
CD45	1.1 ± 0.2	-	1.5 ± 0.3	-

**Note**: Phenotype: fluorescence intensity (MFI) ratio < 2: - (Negative); 2-2.2: +/- (Borderline); 2.2-10: + (Moderately Positive); 11-20: ++ (Sufficiently Positive); 21-40: +++ (Highly positive); > 41: ++++ (Strongly positive). *Change in phenotype.

**Table 2:** Top KEGG pathways regulated by 16 differentially expressed miRNAs in treated differentiated hAFSCs.

**KEGG Pathway**	***P*-value**	**Gene Count**	**miRNAs Count**
Fatty acid metabolism (hsa01212)	3.594x10^-8^	18	8
Fatty acid biosynthesis (hsa00061)	3.150x10^-7^	4	5
Cell cycle (hsa04110)	1.259x10^-6^	59	11
Fatty acid elongation (hsa00062)	1.389x10^-6^	10	3
Fatty acid degradation (hsa00071)	4.152x10^-5^	16	7
Adherens junction (hsa04520)	5.079x10^-5^	34	11
Pathways in cancer (hsa05200)	1.536x10^-4^	143	11
TGF-beta signaling pathway (hsa04350)	3.509x10^-4^	30	11
Signaling pathways regulating pluripotency of stem cells (hsa04550)	3.509x10^-4^	56	11
mTOR signaling pathway (hsa04150)	2.973x10^-3^	29	11
Sphingolipid metabolism (hsa00600)	0.0158	18	8
Sphingolipid signaling pathway (hsa04071)	0.0218	45	11

## Data Availability

The datasets analyzed during the current research are available from the corresponding author [PB] upon reasonable request.

## LIST OF ABBREVIATIONS

ADHD: Attention Deficit Hyperactivity Disorder
C-Kit: KIT Proto-oncogene, Receptor Tyrosine Kinase
EDTA: Ethylenediaminetetraacetic Acid
FABP4: Fatty Acid Binding Protein 4
GAPDH: Glyceraldehyde-3-phosphate Dehydrogenase
hAFSCs: Human Amniotic Fluid Stem Cells
iPSCs: Pluripotent Stem Cells
LPL: Lipoprotein Lipase
m6A: N6-methyladenosine
NANOG: Nanog Homeobox
NIC: Nicotine
Oct-4: POU Class 5 Homeobox 1
PBS: Phosphate-buffered Saline
PPARG: Peroxisome Proliferator-activated Receptor Gamma
SOX2: SRY-box Transcription Factor 2
