# Supplementary material for: Nicotine-induced Genetic and Epigenetic Modifications in Primary Human Amniotic Fluid Stem Cells
Source: Curr Pharm Des. 2024 Jun 11;30(25):1995–2006. doi: 10.2174/0113816128305232240607084420 (PMC11348467; doi:10.2174/0113816128305232240607084420)
Supplement: Supplementary file 1 [file CPD-30-1995_SD1.pdf]

Supplementary Material

Nicotine-induced Genetic and Epigenetic Modifications in Primary Human Amniotic Fluid Stem Cells

Prabin Upadhyaya[1,#], Cristina Milillo[1,2,#], Annalisa Bruno[1,3], Federico Anaclerio[1,2], Carlotta Buccolini[1,2], Anastasia Dell'Elice[1,2], Ilaria Angilletta[1,2], Marco Gatta[1,3], Patrizia Ballerini[1,3,*] and Ivana Antonucci[1,2]

[1]Center for Advanced Studies and Technology (CAST), "G. d'Annunzio" University of Chieti-Pescara, 66100 Chieti, Italy; [2]Department of Psychological, Health and Territorial Sciences, "G. d'Annunzio" University of Chieti-Pescara, 66100 Chieti, Italy; [3]Department of Innovative Technologies in Medicine & Dentistry, "G. d'Annunzio" University of Chieti-Pescara, 66100 Chieti, Italy

1873-4286/24 $65.00+.00 © 2024 Bentham Science Publishers

Table S1. Primers used for qPCR analysis.

Gene name	Forward Primer (5' -> 3')		Reverse Primer (5' -> 3')	
	Sequence	-mer	Sequence	-mer
C-Kit (KIT proto-oncogene, receptor tyrosine kinase)	CCACACCCTGTTCACTCCTT	20	TTCTGGGAAACTCCCAT TTG	20
Oct-4 (POU class 5 homeobox 1)	CTTGCTGCAGAAGTGGGTGGAGGAA	25	CTGCAGTGTGGGTTTCGGGCA	21
SOX2 (SRY-box transcription factor 2)	TTGCTGCCTCTTTAAGACTAGGA	23	CTGGGGCTCAAACTTCTCTC	20
NANOG (Nanog homeobox)	AGATGCCTCACACGGAGACT	20	GTTTGCCTTTGGGACTGGTG	20
LPL (lipoprotein lipase)	CTCTTGGGATACAGCCTTGG	20	GGG GCT TCT GCA TAC TCAAA	20
PPARG (peroxisome proliferator-activated receptor gamma)	CCAGAAAGCGATTCCTTCAC	20	GAG AGA TCC ACG GAG CTG AT	20
FABP4 (fatty acid binding protein 4)	AACCTTAGATGGGGGTGTCC	20	GTG GAA GTG ACG CCT TTC AT	20
GAPDH (Reference gene)	ACCATCTTCCAGGAGCGAGA	20	AGTGATGGCATGGACTGTGG	20

Table S2. List of antibodies used Immunophenotyping with flow cytometry.

Antibody	Manufacturer
**Primary Conjugated**	
Anti-human CD13-FITC	Ancell (MN, USA)
Anti-human CD44-FITC	
Anti-human CD45-FITC	
Anti-human CD29-PE	
Anti-human CD105-FITC	
Anti-human CD166-FITC	
Anti-human CD14-FITC	Miltenyi Biotec (Bergisch Gladbach, Germany);
Anti-human HLA-DR-PE	Becton Dickinson (San Jose, CA, USA)
Anti-human HLA-ABC- Alexa488	
Anti-human CD90-FITC	
Anti-human CD73-PE	
Anti-human SSEA4-FITC	
Anti-human CD117-APC	
Anti-human CD146-PE	
Anti-human CD144-FITC	Acris Antibodies (Herford, Germany)
Anti-human CD34-PE	Beckman Coulter (Fullerton, CA, USA)
**Primary Unconjugated**	
Mouse anti-human NANOG	ThermoFisher Scientific (MA, USA)
Mouse anti-human Sox2	
Rabbit anti-human OCT-4	
**Secondary Conjugated antibodies to bind against primary unconjugated**	
Alexa Fluor® 488 conjugated goat anti-mouse IgG	ThermoFisher Scientific (MA, USA)
Alexa Fluor® 532 conjugated goat anti-Rabbit IgG (H+L)	

Table S3. Primers and PCR conditions used in pyrosequencing.

Gene Promoter	Forward Primer (5' → 3') [Bio]= Biotinylated at 5' end	Reverse Primer (5' → 3') [Bio]= Biotinylated at 5' end	Sequence primer (5'→3')	CpGs assayed (n)	CpGs Analysed (n)	Amplicon size	Annealing Temp (ºC)
H-19	GGTTTTGGAGG TTAGTGTTTT	[Bio]CTCAACCCCTAA AACTAACTTAACA	TTGTATTATTTTT TTTTTTGAGAGT	5	5	187	64

Table S4. List of analyzed miRNAs.

hsa-miR-4640-5p	hsa-miR-15b-5p	hsa-miR-3126-3p	hsa-miR-423-5p	hsa-miR-505-3p	hsa-miR-6772-3p
hsa-let-7a-2-3p	hsa-miR-16-2-3p	hsa-miR-3126-5p	hsa-miR-424-3p	hsa-miR-505-5p	hsa-miR-6775-3p
hsa-let-7a-3p	hsa-miR-16-5p	hsa-miR-3127-3p	hsa-miR-424-5p	hsa-miR-5090	hsa-miR-6776-3p
hsa-let-7a-5p	hsa-miR-17-3p	hsa-miR-3127-5p	hsa-miR-425-3p	hsa-miR-5091	hsa-miR-6777-3p
hsa-let-7b-3p	hsa-miR-17-5p	hsa-miR-3129-3p	hsa-miR-4254	hsa-miR-5100	hsa-miR-6777-5p
hsa-let-7b-5p	hsa-miR-181a-2-3p	hsa-miR-3129-5p	hsa-miR-425-5p	hsa-miR-514a-3p	hsa-miR-6779-3p
hsa-let-7c-5p	hsa-miR-181a-3p	hsa-miR-3130-3p	hsa-miR-4284	hsa-miR-515-5p	hsa-miR-6780b-3p
hsa-let-7d-3p	hsa-miR-181a-5p	hsa-miR-3130-5p	hsa-miR-429	hsa-miR-5187-5p	hsa-miR-6781-3p
hsa-let-7d-5p	hsa-miR-181b-2-3p	hsa-miR-3135b	hsa-miR-431-3p	hsa-miR-5189-5p	hsa-miR-6784-3p
hsa-let-7e-3p	hsa-miR-181b-3p	hsa-miR-3139	hsa-miR-431-5p	hsa-miR-518b	hsa-miR-6785-3p
hsa-let-7e-5p	hsa-miR-181b-5p	hsa-miR-31-3p	hsa-miR-432-3p	hsa-miR-5190	hsa-miR-6786-3p
hsa-let-7f-1-3p	hsa-miR-181c-3p	hsa-miR-3141	hsa-miR-4324	hsa-miR-532-3p	hsa-miR-6787-3p
hsa-let-7f-2-3p	hsa-miR-181c-5p	hsa-miR-3142	hsa-miR-432-5p	hsa-miR-532-5p	hsa-miR-6787-5p
hsa-let-7f-5p	hsa-miR-181d-5p	hsa-miR-3145-3p	hsa-miR-433-3p	hsa-miR-539-3p	hsa-miR-6788-3p
hsa-let-7g-3p	hsa-miR-182-5p	hsa-miR-3149	hsa-miR-433-5p	hsa-miR-539-5p	hsa-miR-6788-5p
hsa-let-7g-5p	hsa-miR-1827	hsa-miR-3157-3p	hsa-miR-4429	hsa-miR-541-3p	hsa-miR-6789-5p
hsa-let-7i-3p	hsa-miR-183-5p	hsa-miR-3157-5p	hsa-miR-4433b-5p	hsa-miR-541-5p	hsa-miR-6791-3p
hsa-let-7i-5p	hsa-miR-184	hsa-miR-3158-3p	hsa-miR-4435	hsa-miR-542-3p	hsa-miR-6791-5p
hsa-miR-100-3p	hsa-miR-185-3p	hsa-miR-31-5p	hsa-miR-4436a	hsa-miR-542-5p	hsa-miR-6793-3p
hsa-miR-100-5p	hsa-miR-185-5p	hsa-miR-3161	hsa-miR-4436b-3p	hsa-miR-543	hsa-miR-6793-5p
hsa-miR-101-3p	hsa-miR-186-3p	hsa-miR-3168	hsa-miR-4436b-5p	hsa-miR-545-5p	hsa-miR-6796-5p
hsa-miR-101-5p	hsa-miR-186-5p	hsa-miR-3170	hsa-miR-4443	hsa-miR-548a-3p	hsa-miR-6797-3p
hsa-miR-103a-2-5p	hsa-miR-187-3p	hsa-miR-3173-3p	hsa-miR-4448	hsa-miR-548ab	hsa-miR-6798-3p

hsa-miR-103a-3p	hsa-miR-188-3p	hsa-miR-3175	hsa-miR-4449	hsa-miR-548al	hsa-miR-6799-3p
hsa-miR-106a-5p	hsa-miR-188-5p	hsa-miR-3176	hsa-miR-4451	hsa-miR-548av-5p	hsa-miR-6802-3p
hsa-miR-106b-3p	hsa-miR-18a-3p	hsa-miR-3177-3p	hsa-miR-4454	hsa-miR-548ay-3p	hsa-miR-6803-3p
hsa-miR-106b-5p	hsa-miR-18a-5p	hsa-miR-3177-5p	hsa-miR-4458	hsa-miR-548az-5p	hsa-miR-6805-3p
hsa-miR-107	hsa-miR-18b-5p	hsa-miR-3180	hsa-miR-4459	hsa-miR-548ba	hsa-miR-6805-5p
hsa-miR-10a-3p	hsa-miR-1908-3p	hsa-miR-3180-3p	hsa-miR-4461	hsa-miR-548e-3p	hsa-miR-6807-3p
hsa-miR-10a-5p	hsa-miR-1908-5p	hsa-miR-3180-5p	hsa-miR-4463	hsa-miR-548e-5p	hsa-miR-6808-3p
hsa-miR-10b-3p	hsa-miR-1909-3p	hsa-miR-3181	hsa-miR-4466	hsa-miR-548k	hsa-miR-6809-3p
hsa-miR-10b-5p	hsa-miR-1909-5p	hsa-miR-3182	hsa-miR-4473	hsa-miR-548l	hsa-miR-6810-3p
hsa-miR-1179	hsa-miR-190a-3p	hsa-miR-3183	hsa-miR-4474-3p	hsa-miR-548n	hsa-miR-6810-5p
hsa-miR-1180-3p	hsa-miR-190a-5p	hsa-miR-3184-3p	hsa-miR-4478	hsa-miR-548o-3p	hsa-miR-6811-5p
hsa-miR-1180-5p	hsa-miR-190b	hsa-miR-3187-3p	hsa-miR-4479	hsa-miR-548q	hsa-miR-6812-3p
hsa-miR-1185-1-3p	hsa-miR-1910-5p	hsa-miR-3187-5p	hsa-miR-4484	hsa-miR-548s	hsa-miR-6814-5p
hsa-miR-1185-2-3p	hsa-miR-1913	hsa-miR-3188	hsa-miR-4485-3p	hsa-miR-548u	hsa-miR-6815-5p
hsa-miR-1185-5p	hsa-miR-191-3p	hsa-miR-3192-5p	hsa-miR-4485-5p	hsa-miR-549a	hsa-miR-6816-3p
hsa-miR-1193	hsa-miR-1914-5p	hsa-miR-3193	hsa-miR-4489	hsa-miR-550a-3-5p	hsa-miR-6817-3p
hsa-miR-1197	hsa-miR-1915-3p	hsa-miR-3195	hsa-miR-4498	hsa-miR-550a-3p	hsa-miR-6818-3p
hsa-miR-1224-3p	hsa-miR-1915-5p	hsa-miR-3200-3p	hsa-miR-449a	hsa-miR-550a-5p	hsa-miR-6818-5p
hsa-miR-1224-5p	hsa-miR-191-5p	hsa-miR-320a	hsa-miR-449c-5p	hsa-miR-551b-3p	hsa-miR-6819-3p
hsa-miR-1225-3p	hsa-miR-192-3p	hsa-miR-320b	hsa-miR-4500	hsa-miR-551b-5p	hsa-miR-6819-5p
hsa-miR-122-5p	hsa-miR-192-5p	hsa-miR-320c	hsa-miR-450a-2-3p	hsa-miR-5579-3p	hsa-miR-6820-3p
hsa-miR-1226-3p	hsa-miR-193a-3p	hsa-miR-320d	hsa-miR-450a-5p	hsa-miR-5581-3p	hsa-miR-6820-5p
hsa-miR-1226-5p	hsa-miR-193a-5p	hsa-miR-320e	hsa-miR-450b-5p	hsa-miR-5585-5p	hsa-miR-6824-3p
hsa-miR-1227-3p	hsa-miR-193b-3p	hsa-miR-323a-3p	hsa-miR-4510	hsa-miR-5587-3p	hsa-miR-6825-5p
hsa-miR-1228-3p	hsa-miR-193b-5p	hsa-miR-323a-5p	hsa-miR-4511	hsa-miR-561-5p	hsa-miR-6827-3p
hsa-miR-1228-5p	hsa-miR-194-3p	hsa-miR-323b-3p	hsa-miR-4512	hsa-miR-5683	hsa-miR-6829-5p
hsa-miR-1229-3p	hsa-miR-194-5p	hsa-miR-32-3p	hsa-miR-451a	hsa-miR-5690	hsa-miR-6830-3p
hsa-miR-1233-3p	hsa-miR-195-5p	hsa-miR-324-3p	hsa-miR-4521	hsa-miR-5695	hsa-miR-6831-3p
hsa-miR-1233-5p	hsa-miR-196a-3p	hsa-miR-324-5p	hsa-miR-452-5p	hsa-miR-5697	hsa-miR-6836-3p

hsa-miR-1234-3p	hsa-miR-196a-5p	hsa-miR-32-5p	hsa-miR-4526	hsa-miR-5699-5p	hsa-miR-6836-5p
hsa-miR-1236-5p	hsa-miR-196b-3p	hsa-miR-326	hsa-miR-454-3p	hsa-miR-5701	hsa-miR-6837-3p
hsa-miR-1237-3p	hsa-miR-196b-5p	hsa-miR-328-3p	hsa-miR-454-5p	hsa-miR-570-3p	hsa-miR-6838-5p
hsa-miR-1237-5p	hsa-miR-1973	hsa-miR-329-3p	hsa-miR-455-3p	hsa-miR-573	hsa-miR-6840-5p
hsa-miR-1243	hsa-miR-197-3p	hsa-miR-329-5p	hsa-miR-455-5p	hsa-miR-574-3p	hsa-miR-6842-3p
hsa-miR-1244	hsa-miR-197-5p	hsa-miR-330-3p	hsa-miR-4636	hsa-miR-576-3p	hsa-miR-6844
hsa-miR-1245a	hsa-miR-1976	hsa-miR-330-5p	hsa-miR-4638-3p	hsa-miR-576-5p	hsa-miR-6845-3p
hsa-miR-1246	hsa-miR-199a-3p	hsa-miR-331-3p	hsa-miR-4638-5p	hsa-miR-577	hsa-miR-6845-5p
hsa-miR-1247-5p	hsa-miR-199a-5p	hsa-miR-331-5p	hsa-miR-4640-3p	hsa-miR-579-5p	hsa-miR-6847-5p
hsa-miR-1248	hsa-miR-199b-5p	hsa-miR-335-3p	hsa-miR-4645-3p	hsa-miR-580-3p	hsa-miR-6848-5p
hsa-miR-1249-3p	hsa-miR-19a-3p	hsa-miR-335-5p	hsa-miR-4646-3p	hsa-miR-582-3p	hsa-miR-6849-3p
hsa-miR-1249-5p	hsa-miR-19a-5p	hsa-miR-337-3p	hsa-miR-4647	hsa-miR-582-5p	hsa-miR-6851-3p
hsa-miR-1252-5p	hsa-miR-19b-3p	hsa-miR-337-5p	hsa-miR-4649-3p	hsa-miR-584-5p	hsa-miR-6851-5p
hsa-miR-1255a	hsa-miR-200a-3p	hsa-miR-338-3p	hsa-miR-4649-5p	hsa-miR-585-3p	hsa-miR-6852-5p
hsa-miR-125a-3p	hsa-miR-200a-5p	hsa-miR-339-3p	hsa-miR-4651	hsa-miR-585-5p	hsa-miR-6854-5p
hsa-miR-125a-5p	hsa-miR-200b-3p	hsa-miR-339-5p	hsa-miR-4655-5p	hsa-miR-588	hsa-miR-6855-5p
hsa-miR-125b-1-3p	hsa-miR-200b-5p	hsa-miR-33a-3p	hsa-miR-466	hsa-miR-589-3p	hsa-miR-6857-3p
hsa-miR-125b-2-3p	hsa-miR-200c-3p	hsa-miR-33a-5p	hsa-miR-4660	hsa-miR-589-5p	hsa-miR-6857-5p
hsa-miR-125b-5p	hsa-miR-203a-3p	hsa-miR-33b-3p	hsa-miR-4661-5p	hsa-miR-590-3p	hsa-miR-6858-3p
hsa-miR-1260a	hsa-miR-204-3p	hsa-miR-33b-5p	hsa-miR-4665-5p	hsa-miR-590-5p	hsa-miR-6858-5p
hsa-miR-1260b	hsa-miR-204-5p	hsa-miR-340-3p	hsa-miR-4666a-5p	hsa-miR-597-3p	hsa-miR-6859-3p
hsa-miR-1262	hsa-miR-205-5p	hsa-miR-340-5p	hsa-miR-4667-3p	hsa-miR-598-3p	hsa-miR-6859-5p
hsa-miR-126-3p	hsa-miR-20a-3p	hsa-miR-342-3p	hsa-miR-4667-5p	hsa-miR-598-5p	hsa-miR-6860
hsa-miR-126-5p	hsa-miR-20a-5p	hsa-miR-342-5p	hsa-miR-4668-5p	hsa-miR-605-5p	hsa-miR-6865-3p
hsa-miR-1270	hsa-miR-20b-5p	hsa-miR-345-5p	hsa-miR-4671-5p	hsa-miR-612	hsa-miR-6868-3p
hsa-miR-1271-5p	hsa-miR-210-3p	hsa-miR-34a-3p	hsa-miR-4677-3p	hsa-miR-6129	hsa-miR-6871-5p
hsa-miR-1273d	hsa-miR-210-5p	hsa-miR-34a-5p	hsa-miR-4677-5p	hsa-miR-6131	hsa-miR-6873-5p
hsa-miR-127-3p	hsa-miR-2110	hsa-miR-34b-3p	hsa-miR-4685-3p	hsa-miR-6132	hsa-miR-6875-5p
hsa-miR-1275	hsa-miR-2114-3p	hsa-miR-34b-5p	hsa-miR-4687-3p	hsa-miR-6134	hsa-miR-6876-5p

hsa-miR-127-5p	hsa-miR-2114-5p	hsa-miR-34c-3p	hsa-miR-4687-5p	hsa-miR-615-3p	hsa-miR-6877-5p
hsa-miR-1276	hsa-miR-2116-3p	hsa-miR-34c-5p	hsa-miR-4688	hsa-miR-616-3p	hsa-miR-6879-3p
hsa-miR-1277-3p	hsa-miR-212-3p	hsa-miR-3605-3p	hsa-miR-4700-3p	hsa-miR-616-5p	hsa-miR-6880-3p
hsa-miR-1277-5p	hsa-miR-212-5p	hsa-miR-3605-5p	hsa-miR-4707-3p	hsa-miR-618	hsa-miR-6881-3p
hsa-miR-1278	hsa-miR-21-3p	hsa-miR-3607-3p	hsa-miR-4709-3p	hsa-miR-625-3p	hsa-miR-6882-5p
hsa-miR-128-1-5p	hsa-miR-214-3p	hsa-miR-3607-5p	hsa-miR-4709-5p	hsa-miR-625-5p	hsa-miR-6885-5p
hsa-miR-128-2-5p	hsa-miR-215-5p	hsa-miR-3609	hsa-miR-4712-3p	hsa-miR-627-3p	hsa-miR-6886-3p
hsa-miR-1283	hsa-miR-21-5p	hsa-miR-3611	hsa-miR-4714-3p	hsa-miR-627-5p	hsa-miR-6886-5p
hsa-miR-128-3p	hsa-miR-216a-3p	hsa-miR-3613-5p	hsa-miR-4716-3p	hsa-miR-628-3p	hsa-miR-6888-3p
hsa-miR-1284	hsa-miR-216a-5p	hsa-miR-361-3p	hsa-miR-4717-3p	hsa-miR-628-5p	hsa-miR-6889-3p
hsa-miR-1286	hsa-miR-217	hsa-miR-3615	hsa-miR-4723-3p	hsa-miR-629-5p	hsa-miR-6891-5p
hsa-miR-1287-5p	hsa-miR-218-2-3p	hsa-miR-361-5p	hsa-miR-4725-3p	hsa-miR-636	hsa-miR-6892-5p
hsa-miR-1291	hsa-miR-218-5p	hsa-miR-3620-3p	hsa-miR-4725-5p	hsa-miR-641	hsa-miR-6894-3p
hsa-miR-1292-3p	hsa-miR-219a-1-3p	hsa-miR-3620-5p	hsa-miR-4728-3p	hsa-miR-6500-3p	hsa-miR-6894-5p
hsa-miR-129-2-3p	hsa-miR-219a-5p	hsa-miR-3622a-3p	hsa-miR-4728-5p	hsa-miR-6505-3p	hsa-miR-6895-3p
hsa-miR-1292-5p	hsa-miR-219b-3p	hsa-miR-3622a-5p	hsa-miR-4737	hsa-miR-6508-5p	hsa-miR-708-3p
hsa-miR-1293	hsa-miR-219b-5p	hsa-miR-362-3p	hsa-miR-4741	hsa-miR-6509-5p	hsa-miR-708-5p
hsa-miR-1294	hsa-miR-221-3p	hsa-miR-362-5p	hsa-miR-4742-3p	hsa-miR-6511a-3p	hsa-miR-7106-3p
hsa-miR-129-5p	hsa-miR-221-5p	hsa-miR-3651	hsa-miR-4742-5p	hsa-miR-6511a-5p	hsa-miR-7106-5p
hsa-miR-1296-5p	hsa-miR-222-3p	hsa-miR-3653-3p	hsa-miR-4743-3p	hsa-miR-6511b-3p	hsa-miR-7108-3p
hsa-miR-1299	hsa-miR-222-5p	hsa-miR-3653-5p	hsa-miR-4743-5p	hsa-miR-6511b-5p	hsa-miR-7108-5p
hsa-miR-1301-3p	hsa-miR-223-3p	hsa-miR-365a-3p	hsa-miR-4745-3p	hsa-miR-6512-5p	hsa-miR-7109-3p
hsa-miR-1302	hsa-miR-22-3p	hsa-miR-365a-5p	hsa-miR-4745-5p	hsa-miR-6513-5p	hsa-miR-7110-3p
hsa-miR-1304-3p	hsa-miR-224-3p	hsa-miR-365b-5p	hsa-miR-4746-5p	hsa-miR-6514-5p	hsa-miR-7110-5p
hsa-miR-1304-5p	hsa-miR-224-5p	hsa-miR-3661	hsa-miR-4749-3p	hsa-miR-6515-3p	hsa-miR-7111-3p
hsa-miR-1305	hsa-miR-22-5p	hsa-miR-3677-3p	hsa-miR-4750-5p	hsa-miR-6515-5p	hsa-miR-7111-5p
hsa-miR-1306-3p	hsa-miR-2277-3p	hsa-miR-3679-5p	hsa-miR-4757-3p	hsa-miR-651-5p	hsa-miR-7113-5p
hsa-miR-1306-5p	hsa-miR-2277-5p	hsa-miR-3685	hsa-miR-4758-3p	hsa-miR-6516-3p	hsa-miR-7114-3p
hsa-miR-1307-3p	hsa-miR-2355-3p	hsa-miR-3687	hsa-miR-4761-3p	hsa-miR-6516-5p	hsa-miR-7-1-3p

hsa-miR-1307-5p	hsa-miR-2355-5p	hsa-miR-3691-5p	hsa-miR-4762-5p	hsa-miR-652-3p	hsa-miR-718
hsa-miR-130a-3p	hsa-miR-2392	hsa-miR-369-3p	hsa-miR-4766-5p	hsa-miR-652-5p	hsa-miR-744-3p
hsa-miR-130b-3p	hsa-miR-23a-3p	hsa-miR-369-5p	hsa-miR-4767	hsa-miR-653-3p	hsa-miR-744-5p
hsa-miR-130b-5p	hsa-miR-23a-5p	hsa-miR-370-3p	hsa-miR-4775	hsa-miR-653-5p	hsa-miR-758-3p
hsa-miR-1322	hsa-miR-23b-3p	hsa-miR-370-5p	hsa-miR-4783-3p	hsa-miR-654-3p	hsa-miR-758-5p
hsa-miR-1323	hsa-miR-23b-5p	hsa-miR-374a-3p	hsa-miR-4787-3p	hsa-miR-654-5p	hsa-miR-7-5p
hsa-miR-132-3p	hsa-miR-23c	hsa-miR-374a-5p	hsa-miR-4787-5p	hsa-miR-655-3p	hsa-miR-760
hsa-miR-132-5p	hsa-miR-24-1-5p	hsa-miR-374b-3p	hsa-miR-4788	hsa-miR-656-3p	hsa-miR-7641
hsa-miR-133a-3p	hsa-miR-24-2-5p	hsa-miR-374b-5p	hsa-miR-4791	hsa-miR-659-5p	hsa-miR-765
hsa-miR-1343-3p	hsa-miR-24-3p	hsa-miR-374c-5p	hsa-miR-4792	hsa-miR-660-3p	hsa-miR-766-3p
hsa-miR-134-3p	hsa-miR-2467-5p	hsa-miR-375	hsa-miR-4797-3p	hsa-miR-660-5p	hsa-miR-769-3p
hsa-miR-134-5p	hsa-miR-25-3p	hsa-miR-376a-2-5p	hsa-miR-4802-3p	hsa-miR-664a-3p	hsa-miR-769-5p
hsa-miR-135a-5p	hsa-miR-25-5p	hsa-miR-376a-3p	hsa-miR-4804-5p	hsa-miR-664a-5p	hsa-miR-7703
hsa-miR-135b-5p	hsa-miR-2682-3p	hsa-miR-376a-5p	hsa-miR-483-3p	hsa-miR-664b-3p	hsa-miR-7704
hsa-miR-136-3p	hsa-miR-2682-5p	hsa-miR-376b-3p	hsa-miR-483-5p	hsa-miR-664b-5p	hsa-miR-7705
hsa-miR-136-5p	hsa-miR-26a-1-3p	hsa-miR-376b-5p	hsa-miR-484	hsa-miR-665	hsa-miR-770-5p
hsa-miR-137	hsa-miR-26a-2-3p	hsa-miR-376c-3p	hsa-miR-485-3p	hsa-miR-668-3p	hsa-miR-7706
hsa-miR-138-1-3p	hsa-miR-26a-5p	hsa-miR-376c-5p	hsa-miR-485-5p	hsa-miR-671-3p	hsa-miR-7845-5p
hsa-miR-138-2-3p	hsa-miR-26b-3p	hsa-miR-377-3p	hsa-miR-486-3p	hsa-miR-671-5p	hsa-miR-7854-3p
hsa-miR-138-5p	hsa-miR-26b-5p	hsa-miR-377-5p	hsa-miR-486-5p	hsa-miR-6716-3p	hsa-miR-7974
hsa-miR-139-3p	hsa-miR-27a-3p	hsa-miR-378a-3p	hsa-miR-487a-3p	hsa-miR-6721-5p	hsa-miR-7975
hsa-miR-139-5p	hsa-miR-27a-5p	hsa-miR-378a-5p	hsa-miR-487a-5p	hsa-miR-6723-5p	hsa-miR-7977
hsa-miR-1-3p	hsa-miR-27b-3p	hsa-miR-378c	hsa-miR-487b-3p	hsa-miR-6726-3p	hsa-miR-8072
hsa-miR-140-3p	hsa-miR-27b-5p	hsa-miR-378d	hsa-miR-487b-5p	hsa-miR-6727-3p	hsa-miR-873-3p
hsa-miR-140-5p	hsa-miR-28-3p	hsa-miR-378e	hsa-miR-489-3p	hsa-miR-6727-5p	hsa-miR-873-5p
hsa-miR-141-3p	hsa-miR-28-5p	hsa-miR-378f	hsa-miR-490-3p	hsa-miR-6729-3p	hsa-miR-874-3p
hsa-miR-141-5p	hsa-miR-296-3p	hsa-miR-378g	hsa-miR-490-5p	hsa-miR-6730-5p	hsa-miR-874-5p
hsa-miR-142-3p	hsa-miR-296-5p	hsa-miR-378i	hsa-miR-491-3p	hsa-miR-6732-3p	hsa-miR-876-3p
hsa-miR-142-5p	hsa-miR-299-3p	hsa-miR-379-3p	hsa-miR-491-5p	hsa-miR-6734-5p	hsa-miR-877-3p

hsa-miR-143-3p	hsa-miR-299-5p	hsa-miR-379-5p	hsa-miR-493-3p	hsa-miR-6735-3p	hsa-miR-877-5p
hsa-miR-143-5p	hsa-miR-29a-3p	hsa-miR-380-3p	hsa-miR-493-5p	hsa-miR-6735-5p	hsa-miR-885-5p
hsa-miR-144-3p	hsa-miR-29a-5p	hsa-miR-380-5p	hsa-miR-494-3p	hsa-miR-6736-3p	hsa-miR-887-3p
hsa-miR-144-5p	hsa-miR-29b-1-5p	hsa-miR-381-3p	hsa-miR-494-5p	hsa-miR-6736-5p	hsa-miR-887-5p
hsa-miR-145-3p	hsa-miR-29b-2-5p	hsa-miR-381-5p	hsa-miR-495-3p	hsa-miR-6737-3p	hsa-miR-889-3p
hsa-miR-145-5p	hsa-miR-29b-3p	hsa-miR-382-3p	hsa-miR-496	hsa-miR-6739-3p	hsa-miR-889-5p
hsa-miR-1468-5p	hsa-miR-29c-3p	hsa-miR-382-5p	hsa-miR-497-5p	hsa-miR-6740-5p	hsa-miR-921
hsa-miR-146a-3p	hsa-miR-29c-5p	hsa-miR-383-5p	hsa-miR-4999-3p	hsa-miR-6741-3p	hsa-miR-922
hsa-miR-146a-5p	hsa-miR-301a-3p	hsa-miR-3909	hsa-miR-499a-5p	hsa-miR-6742-3p	hsa-miR-92a-1-5p
hsa-miR-146b-3p	hsa-miR-301b-3p	hsa-miR-3912-3p	hsa-miR-5000-3p	hsa-miR-6743-3p	hsa-miR-92a-3p
hsa-miR-146b-5p	hsa-miR-302a-3p	hsa-miR-3913-3p	hsa-miR-5001-3p	hsa-miR-6746-3p	hsa-miR-92b-3p
hsa-miR-147b	hsa-miR-302a-5p	hsa-miR-3913-5p	hsa-miR-5001-5p	hsa-miR-6747-3p	hsa-miR-92b-5p
hsa-miR-148a-3p	hsa-miR-302c-3p	hsa-miR-3916	hsa-miR-5002-5p	hsa-miR-6749-3p	hsa-miR-93-3p
hsa-miR-148a-5p	hsa-miR-3065-3p	hsa-miR-3917	hsa-miR-5006-3p	hsa-miR-6750-3p	hsa-miR-935
hsa-miR-148b-3p	hsa-miR-3065-5p	hsa-miR-3928-3p	hsa-miR-5008-3p	hsa-miR-6751-5p	hsa-miR-93-5p
hsa-miR-148b-5p	hsa-miR-30a-3p	hsa-miR-3929	hsa-miR-5009-5p	hsa-miR-6752-3p	hsa-miR-937-3p
hsa-miR-149-3p	hsa-miR-30a-5p	hsa-miR-3940-3p	hsa-miR-500a-3p	hsa-miR-6752-5p	hsa-miR-937-5p
hsa-miR-149-5p	hsa-miR-30b-3p	hsa-miR-3940-5p	hsa-miR-500a-5p	hsa-miR-6753-3p	hsa-miR-939-3p
hsa-miR-150-5p	hsa-miR-30b-5p	hsa-miR-3943	hsa-miR-500b-3p	hsa-miR-675-3p	hsa-miR-939-5p
hsa-miR-151a-3p	hsa-miR-30c-1-3p	hsa-miR-3944-3p	hsa-miR-500b-5p	hsa-miR-6754-3p	hsa-miR-940
hsa-miR-151a-5p	hsa-miR-30c-2-3p	hsa-miR-409-3p	hsa-miR-5010-3p	hsa-miR-675-5p	hsa-miR-941
hsa-miR-151b	hsa-miR-30c-5p	hsa-miR-409-5p	hsa-miR-5010-5p	hsa-miR-6756-3p	hsa-miR-942-5p
hsa-miR-152-3p	hsa-miR-30d-3p	hsa-miR-410-3p	hsa-miR-501-3p	hsa-miR-6757-3p	hsa-miR-943
hsa-miR-152-5p	hsa-miR-30d-5p	hsa-miR-410-5p	hsa-miR-501-5p	hsa-miR-6762-3p	hsa-miR-944
hsa-miR-153-3p	hsa-miR-30e-3p	hsa-miR-411-3p	hsa-miR-502-3p	hsa-miR-6763-3p	hsa-miR-95-3p
hsa-miR-1538	hsa-miR-30e-5p	hsa-miR-411-5p	hsa-miR-502-5p	hsa-miR-6763-5p	hsa-miR-95-5p
hsa-miR-154-5p	hsa-miR-3117-3p	hsa-miR-412-3p	hsa-miR-503-3p	hsa-miR-6767-5p	hsa-miR-9-5p
hsa-miR-155-5p	hsa-miR-3120-3p	hsa-miR-412-5p	hsa-miR-503-5p	hsa-miR-6769b-3p	hsa-miR-96-5p
hsa-miR-15a-5p	hsa-miR-3123	hsa-miR-421	hsa-miR-504-3p	hsa-miR-6770-3p	hsa-miR-98-3p

hsa-miR-15b-3p	hsa-miR-3124-5p	hsa-miR-423-3p	hsa-miR-504-5p	hsa-miR-6770-5p	hsa-miR-98-5p
					hsa-miR-99a-3p
					hsa-miR-99a-5p
					hsa-miR-99b-3p
					hsa-miR-99b-5p

Table S5. Very highly expressed miRNAs

hsa-miR-21-5p	hsa-miR-143-3p	hsa-miR-10a-5p	hsa-miR-125b-5p	hsa-miR-23b-3p	hsa-miR-30c-5p
hsa-miR-22-3p	hsa-miR-27b-3p	hsa-miR-191-5p	hsa-miR-100-5p	hsa-miR-99b-5p	hsa-miR-31-5p
hsa-miR-10b-5p	hsa-let-7a-5p	hsa-let-7i-5p	hsa-miR-125a-5p	hsa-miR-30d-5p	hsa-let-7g-5p
hsa-miR-181a-5p	hsa-let-7f-5p	hsa-miR-92a-3p	hsa-miR-320a	hsa-miR-30e-5p	
hsa-miR-30a-5p	hsa-miR-26a-5p	hsa-miR-21-3p	hsa-miR-181b-5p	hsa-let-7e-5p	

Table S6. Highly expressed miRNAs

hsa-let-7b-5p	hsa-miR-335-5p	hsa-miR-181a-2-3p	hsa-miR-7977	hsa-miR-654-3p	hsa-miR-107
hsa-miR-151a-3p	hsa-let-7d-5p	hsa-miR-423-3p	hsa-miR-28-3p	hsa-miR-424-5p	hsa-miR-152-3p
hsa-miR-186-5p	hsa-miR-26b-5p	hsa-miR-148b-3p	hsa-miR-181c-5p	hsa-miR-19b-3p	hsa-miR-148a-3p
hsa-miR-103a-3p	hsa-miR-92b-3p	hsa-miR-23a-3p	hsa-miR-197-3p	hsa-miR-484	hsa-miR-181a-3p
hsa-miR-221-3p	hsa-miR-27a-3p	hsa-miR-574-3p	hsa-miR-1260b	hsa-miR-30a-3p	hsa-miR-301a-3p
hsa-miR-151a-5p	hsa-miR-29a-3p	hsa-miR-744-5p	hsa-miR-410-3p	hsa-miR-5701	hsa-miR-93-5p
hsa-miR-151b	hsa-miR-222-3p	hsa-miR-140-3p	hsa-miR-30b-5p	hsa-miR-941	hsa-miR-210-3p
hsa-miR-98-5p	hsa-miR-196b-5p	hsa-miR-192-5p	hsa-miR-25-3p	hsa-miR-1246	hsa-miR-146b-5p
hsa-miR-16-5p	hsa-miR-24-3p	hsa-miR-136-3p	hsa-miR-125b-1-3p	hsa-miR-381-3p	hsa-miR-3182
hsa-miR-101-3p	hsa-miR-130a-3p	hsa-miR-423-5p	hsa-miR-1260a	hsa-miR-196a-5p	
hsa-miR-127-3p	hsa-miR-411-5p	hsa-miR-409-3p	hsa-miR-331-3p	hsa-miR-328-3p	

Table S7. Moderately expressed miRNAs.

hsa-miR-182-5p	hsa-miR-17-5p	hsa-miR-374a-5p	hsa-miR-376c-3p	hsa-miR-99a-5p	hsa-miR-561-5p
hsa-miR-28-5p	hsa-miR-134-5p	hsa-miR-489-3p	hsa-miR-30c-2-3p	hsa-miR-382-5p	hsa-miR-424-3p
hsa-miR-340-5p	hsa-miR-20a-5p	hsa-miR-181d-5p	hsa-miR-193a-5p	hsa-miR-671-3p	hsa-miR-194-5p
hsa-miR-769-5p	hsa-miR-432-5p	hsa-miR-493-5p	hsa-miR-486-5p	hsa-let-7a-3p	hsa-miR-5100
hsa-miR-145-5p	hsa-miR-874-3p	hsa-miR-27b-5p	hsa-miR-106b-5p	hsa-miR-376a-5p	hsa-miR-379-5p
hsa-let-7d-3p	hsa-miR-130b-3p	hsa-miR-210-5p	hsa-miR-493-3p	hsa-miR-193a-3p	hsa-miR-452-5p
hsa-miR-203a-3p	hsa-miR-374a-3p	hsa-miR-374b-5p	hsa-miR-190a-5p	hsa-miR-148b-5p	hsa-miR-450a-5p
hsa-miR-653-5p	hsa-miR-136-5p	hsa-miR-19a-3p	hsa-miR-200b-3p	hsa-miR-494-3p	hsa-miR-887-3p
hsa-miR-378a-3p	hsa-miR-542-3p	hsa-miR-146a-5p	hsa-miR-1296-5p	hsa-miR-1275	hsa-miR-425-3p
hsa-miR-1307-5p	hsa-miR-615-3p	hsa-miR-132-3p	hsa-miR-15b-5p	hsa-miR-582-3p	hsa-miR-654-5p
hsa-miR-425-5p	hsa-miR-889-3p	hsa-miR-339-3p	hsa-miR-450b-5p	hsa-miR-29b-3p	hsa-miR-204-5p
hsa-let-7c-5p	hsa-miR-3607-3p	hsa-miR-664a-3p	hsa-miR-15a-5p	hsa-miR-365a-3p	hsa-miR-485-5p
hsa-miR-34a-5p	hsa-miR-149-5p	hsa-miR-128-3p	hsa-miR-126-5p	hsa-miR-455-5p	hsa-miR-589-5p
hsa-miR-1307-3p	hsa-miR-455-3p	hsa-miR-100-3p	hsa-miR-877-5p	hsa-miR-145-3p	hsa-miR-539-3p
hsa-miR-193b-3p	hsa-miR-342-3p	hsa-miR-31-3p	hsa-miR-99b-3p	hsa-miR-3609	hsa-miR-503-5p
hsa-miR-345-5p	hsa-miR-483-5p	hsa-miR-370-3p	hsa-miR-24-2-5p	hsa-miR-106b-3p	hsa-miR-337-3p
hsa-miR-500a-3p	hsa-miR-335-3p	hsa-miR-155-5p	hsa-miR-4461	hsa-miR-369-3p	hsa-let-7f-1-3p
hsa-miR-4284	hsa-miR-138-5p	hsa-miR-487b-3p	hsa-miR-361-3p	hsa-miR-323a-3p	hsa-miR-324-3p
hsa-miR-421	hsa-miR-361-5p	hsa-miR-454-3p	hsa-miR-708-3p	hsa-let-7b-3p	hsa-miR-874-5p
hsa-miR-30e-3p	hsa-miR-532-5p	hsa-miR-324-5p	hsa-miR-181c-3p	hsa-miR-532-3p	hsa-miR-183-5p
hsa-miR-221-5p	hsa-miR-320b	hsa-miR-301b-3p	hsa-miR-3615	hsa-miR-369-5p	hsa-miR-142-5p
hsa-miR-708-5p	hsa-miR-1180-3p	hsa-miR-660-5p	hsa-miR-323b-3p	hsa-miR-483-3p	

Table S8. Low expressed miRNAs.

hsa-miR-652-3p	hsa-miR-30c-1-3p	hsa-miR-431-5p	hsa-miR-656-3p	hsa-miR-590-3p	hsa-miR-3187-3p
hsa-miR-30d-3p	hsa-miR-377-3p	hsa-miR-212-3p	hsa-miR-675-3p	hsa-miR-338-3p	hsa-miR-196b-3p
hsa-let-7e-3p	hsa-miR-382-3p	hsa-miR-4449	hsa-miR-3605-3p	hsa-miR-501-5p	hsa-miR-585-3p
hsa-miR-376a-3p	hsa-miR-1908-5p	hsa-miR-26b-3p	hsa-miR-125b-2-3p	hsa-miR-1343-3p	hsa-miR-1277-5p
hsa-miR-495-3p	hsa-miR-873-5p	hsa-miR-376b-3p	hsa-miR-641	hsa-miR-212-5p	hsa-miR-342-5p
hsa-let-7i-3p	hsa-miR-32-5p	hsa-miR-411-3p	hsa-miR-664b-3p	hsa-miR-2110	hsa-miR-15b-3p
hsa-miR-339-5p	hsa-miR-3651	hsa-miR-330-3p	hsa-miR-760	hsa-miR-200c-3p	hsa-miR-134-3p
hsa-miR-1973	hsa-miR-29a-5p	hsa-miR-126-3p	hsa-miR-548k	hsa-miR-653-3p	hsa-miR-326
hsa-miR-4485-3p	hsa-miR-10b-3p	hsa-miR-505-3p	hsa-miR-3605-5p	hsa-miR-4687-5p	hsa-miR-148a-5p
hsa-miR-1248	hsa-miR-485-3p	hsa-miR-200a-3p	hsa-miR-548av-5p	hsa-miR-92a-1-5p	hsa-miR-6798-3p
hsa-miR-668-3p	hsa-miR-2355-5p	hsa-miR-4775	hsa-miR-7-1-3p	hsa-let-7a-2-3p	hsa-miR-33a-3p
hsa-miR-205-5p	hsa-let-7f-2-3p	hsa-miR-766-3p	hsa-miR-1249-3p	hsa-miR-7114-3p	hsa-miR-4709-5p
hsa-miR-140-5p	hsa-miR-181b-3p	hsa-miR-193b-5p	hsa-miR-378c	hsa-miR-331-5p	hsa-miR-6852-5p
hsa-miR-224-5p	hsa-miR-10a-3p	hsa-miR-664a-5p	hsa-miR-429	hsa-miR-1226-3p	hsa-miR-2277-5p
hsa-miR-3065-5p	hsa-miR-487a-3p	hsa-miR-130b-5p	hsa-miR-3613-5p	hsa-miR-3677-3p	hsa-miR-33b-5p
hsa-miR-18a-5p	hsa-miR-542-5p	hsa-miR-296-5p	hsa-miR-379-3p	hsa-miR-16-2-3p	hsa-miR-5699-5p
hsa-miR-98-3p	hsa-miR-337-5p	hsa-miR-1304-3p	hsa-miR-188-5p	hsa-miR-340-3p	hsa-miR-4466
hsa-miR-501-3p	hsa-miR-152-5p	hsa-miR-133a-3p	hsa-miR-4787-3p	hsa-miR-224-3p	hsa-miR-374b-3p
hsa-miR-873-3p	hsa-miR-299-3p	hsa-miR-4485-5p	hsa-miR-744-3p	hsa-miR-6723-5p	hsa-miR-132-5p
hsa-miR-320c	hsa-miR-412-5p	hsa-miR-23c	hsa-miR-1306-5p	hsa-miR-27a-5p	hsa-miR-409-5p
hsa-miR-3065-3p	hsa-miR-377-5p	hsa-miR-431-3p	hsa-miR-1185-1-3p	hsa-miR-2116-3p	hsa-miR-6842-3p
hsa-miR-127-5p	hsa-miR-218-5p	hsa-miR-582-5p	hsa-miR-539-5p	hsa-miR-659-5p	hsa-miR-935
hsa-miR-34c-5p	hsa-miR-154-5p	hsa-miR-3117-3p	hsa-miR-380-3p	hsa-miR-190b	hsa-miR-769-3p
hsa-miR-598-3p	hsa-miR-23a-5p	hsa-miR-95-3p	hsa-miR-655-3p	hsa-miR-3176	hsa-miR-1228-3p
hsa-miR-7706	hsa-miR-143-5p	hsa-miR-1291	hsa-miR-146b-3p	hsa-miR-584-5p	hsa-miR-629-5p
hsa-miR-137	hsa-miR-490-3p	hsa-miR-93-3p	hsa-miR-6511a-3p	hsa-miR-496	hsa-miR-370-5p
hsa-miR-758-3p	hsa-miR-330-5p	hsa-miR-675-5p	hsa-miR-195-5p	hsa-miR-628-3p	hsa-miR-30b-3p
hsa-miR-185-5p	hsa-miR-329-3p	hsa-miR-199a-3p	hsa-miR-181b-2-3p	hsa-miR-770-5p	hsa-miR-6819-3p

hsa-miR-433-3p	hsa-miR-1301-3p	hsa-miR-4677-3p	hsa-miR-3620-3p	hsa-miR-577	hsa-miR-551b-3p
hsa-miR-502-3p	hsa-miR-1185-5p	hsa-miR-24-1-5p	hsa-miR-454-5p	hsa-miR-576-3p	hsa-miR-887-5p
hsa-miR-940	hsa-miR-196a-3p	hsa-miR-4640-3p	hsa-miR-625-5p	hsa-miR-299-5p	hsa-miR-2682-5p
hsa-miR-4454	hsa-miR-22-5p	hsa-miR-362-5p	hsa-miR-320d	hsa-let-7g-3p	hsa-miR-6511a-5p
hsa-miR-92b-5p	hsa-miR-6511b-3p	hsa-miR-17-3p	hsa-miR-26a-2-3p	hsa-miR-589-3p	hsa-miR-6716-3p
hsa-miR-3653-3p	hsa-miR-628-5p	hsa-miR-215-5p	hsa-miR-33a-5p	hsa-miR-550a-5p	hsa-miR-543
hsa-miR-125a-3p	hsa-miR-141-3p	hsa-miR-1197	hsa-miR-222-5p	hsa-miR-550a-3-5p	hsa-miR-190a-3p
hsa-miR-29c-3p	hsa-miR-3909	hsa-miR-128-1-5p	hsa-miR-671-5p	hsa-miR-451a	hsa-miR-6511b-5p
hsa-miR-101-5p	hsa-miR-3944-3p	hsa-miR-1287-5p			

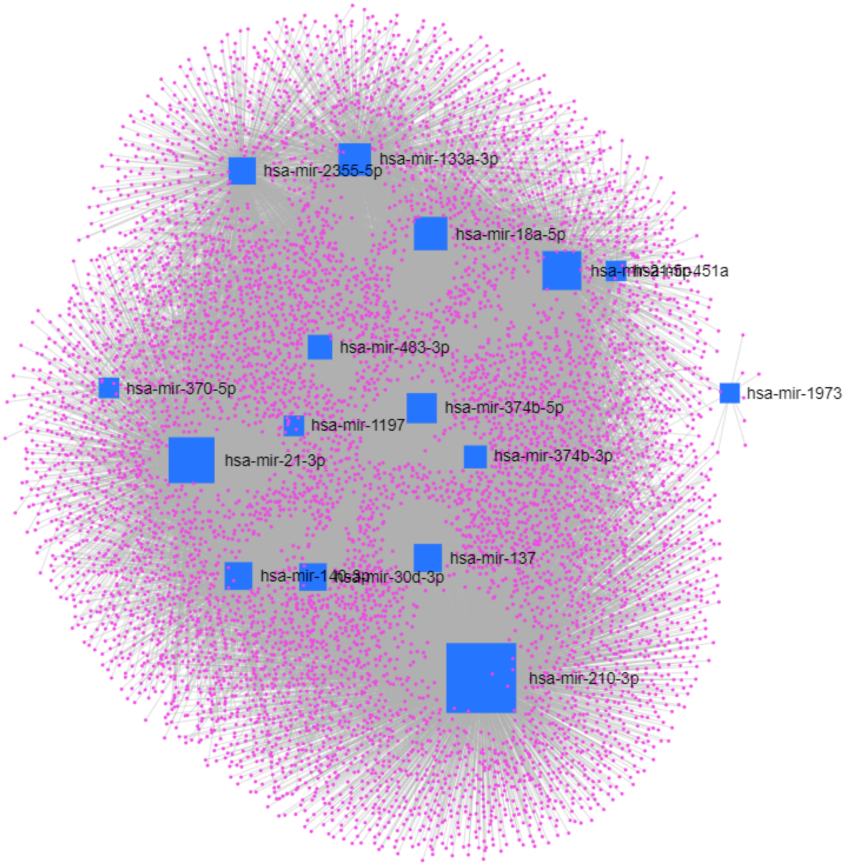
hsa-mir-2355-5p
hsa-mir-133a-3p
hsa-mir-18a-5p
hsa-mir-483-3p
hsa-mir-370-5p
hsa-mir-1973
hsa-mir-374b-5p
hsa-mir-1197
hsa-mir-374b-3p
hsa-mir-21-3p
hsa-mir-137
hsa-mir-210-3p

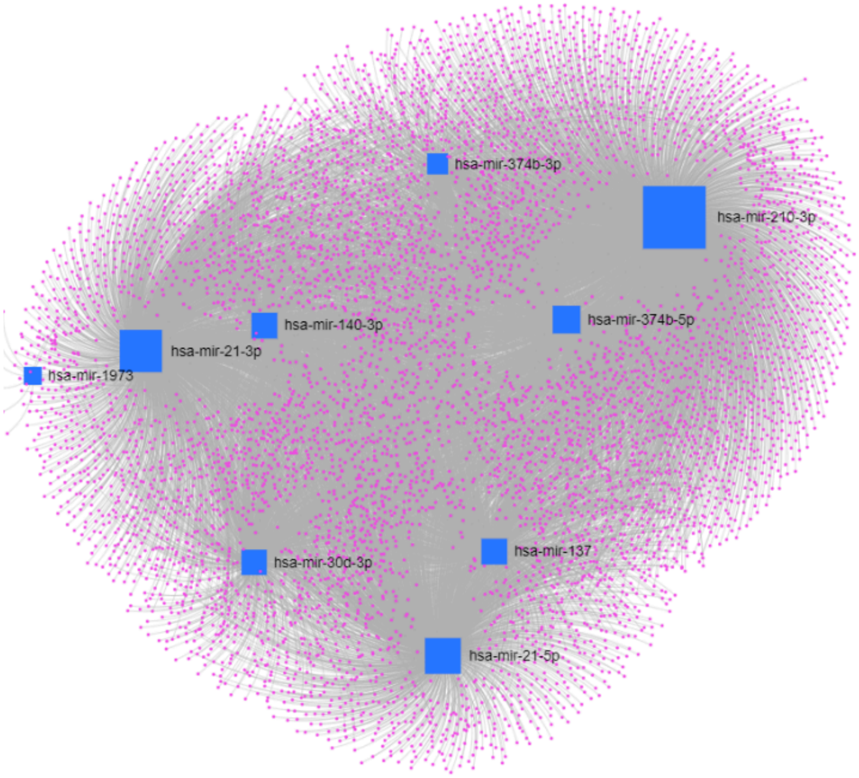
hsa-mir-374b-3p
hsa-mir-210-3p
hsa-mir-140-3p
hsa-mir-374b-5p
hsa-mir-21-3p
hsa-mir-1973
hsa-mir-30d-3p
hsa-mir-137
hsa-mir-21-5p

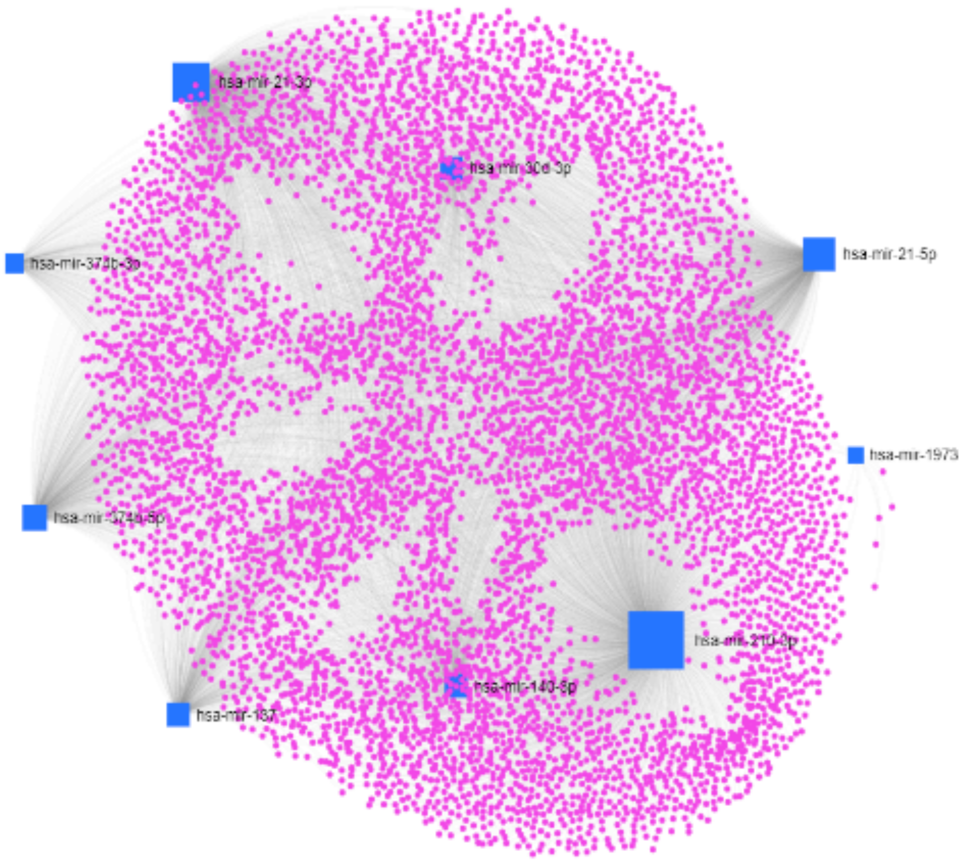

Fig. S1. Functional enrichment of predicted target genes by the bioinformatics tool miRNet. (A) Target gene network for 16 dysregulated miRNAs; (B) Target gene network for upregulated miRNAs; (C) Target gene network for downregulated miRNAs.

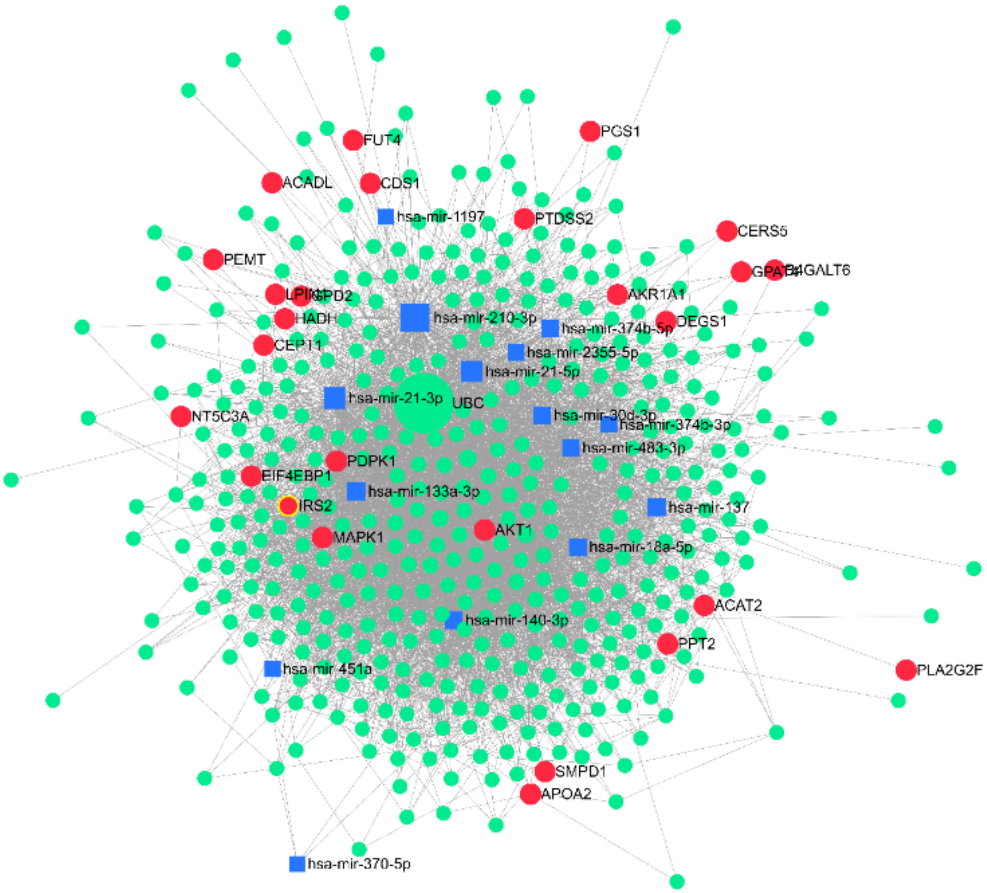

(A)

Functional Category	Adjusted p-value	Genes
Glycerophospholipid metabolism	$5.29 x 10^{-6}$	PEMT<br>PLA2G2F<br>GPD2<br>GPAT4<br>PGS1<br>CEPT1<br>LPIN1<br>CDS1<br>PTDSS2
mTOR signaling pathway	$5.41 x 10^{-3}$	AKT1<br>PDPK1<br>MAPK1<br>EIF4EBP1
Sphingolipid metabolism	$5.41 x 10^{-3}$	SMPD1<br>B4GALT6<br>CERS5<br>DEGS1
Fatty acid metabolism	0.0256	ACAT2<br>HADH<br>ACADL
Glycerolipid metabolism	0.0482	GPAT4<br>AKR1A1<br>LPIN1

(B)

Fig. S2. (A) Network of genes and miRNAs differentially expressed in treated differentiated hAFSCs. The network was generated using miRNet online analysis tool. (B) Results of KEGG functional enrichment analysis for the target genes of differentially expressed miRNAs in treated differentiated hAFSCs

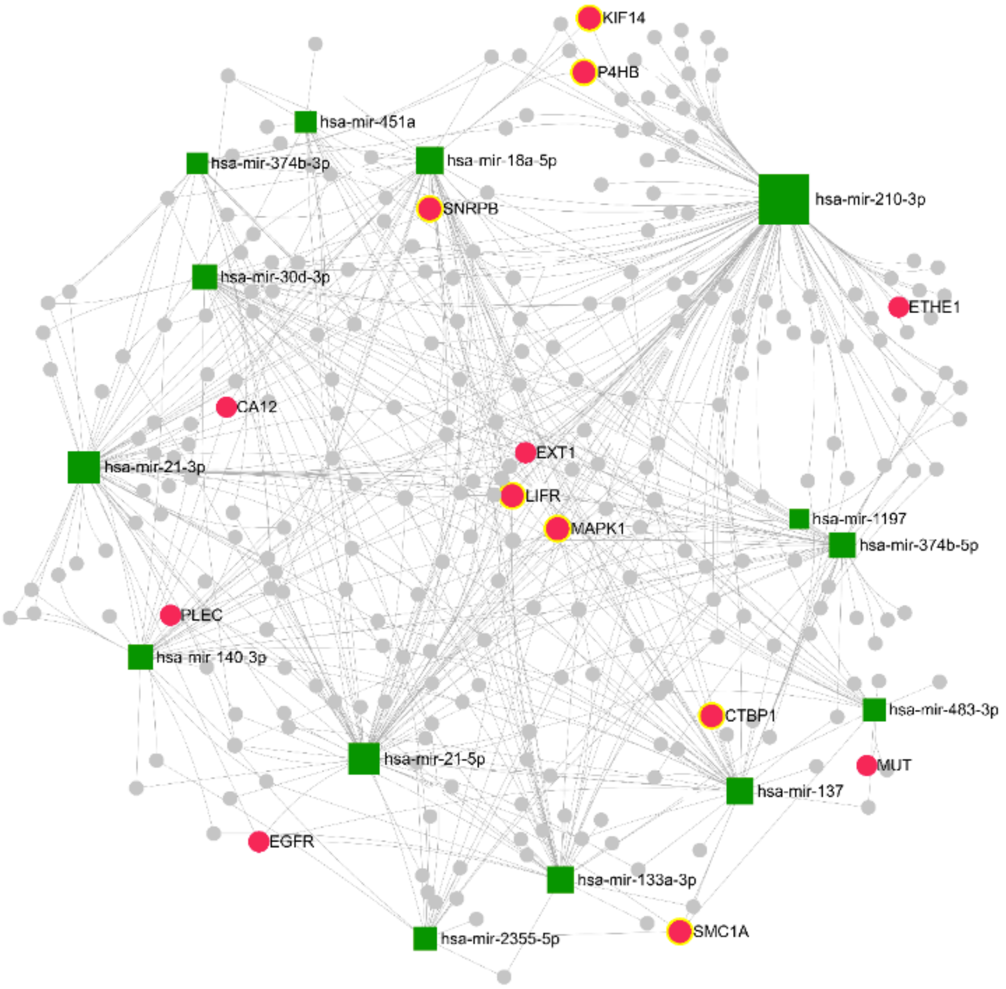

(A)

**Predict diseases**	**Adjusted p-value**	**Genes**
Cognitive delay	$0.02x10^{-6}$	MID1, PAFAH1B1, PCNA, KAT6A, SMC1A, POLR3B, KCNB1, CTBP1, MAPK1, GM2A, MUT, ETHE1, ACADM, MYO5A
Intrauterine retardation	$7.55x10^{-6}$	LIFR, SMC1A, P4HB, CTBP1, MAPK1, KIF14, SNRPB
Infant, Small for Gestational Age	$7.55x10^{-6}$	LIFR, SMC1A, P4HB, CTBP1, MAPK1, KIF14, SNRPB
Fetal Growth Retardation	$9.1x10^{-6}$	LIFR, SMC1A, P4HB, CTBP1, MAPK1, KIF14, SNRPBLIFR, SMC1A, P4HB, CTBP1, MAPK1, KIF14, SNRPB
Failure to gain weight	$1.18x10^{-4}$	CA12, EGFR, SMC1A, CTBP1, MUT, ETHE1, PLEC, EXT1
Premature Birth	$5.44x10^{-3}$	SMC1A, MAPK1, PLEC

(B)

Fig. S3. (A) Network of disease associated miRNA–target gene using Dissent database. (B) Disease-related miRNA–target gene obtained from DisGeNet database.
